# Molecular and clinical determinants of response and resistance to rucaparib for recurrent ovarian cancer treatment in ARIEL2 (Parts 1 and 2)

**DOI:** 10.1038/s41467-021-22582-6

**Published:** 2021-05-03

**Authors:** Elizabeth M. Swisher, Tanya T. Kwan, Amit M. Oza, Anna V. Tinker, Isabelle Ray-Coquard, Ana Oaknin, Robert L. Coleman, Carol Aghajanian, Gottfried E. Konecny, David M. O’Malley, Alexandra Leary, Diane Provencher, Stephen Welch, Lee-may Chen, Andrea E. Wahner Hendrickson, Ling Ma, Prafull Ghatage, Rebecca S. Kristeleit, Oliver Dorigo, Ashan Musafer, Scott H. Kaufmann, Julia A. Elvin, Douglas I. Lin, Setsuko K. Chambers, Erin Dominy, Lan-Thanh Vo, Sandra Goble, Lara Maloney, Heidi Giordano, Thomas Harding, Alexander Dobrovic, Clare L. Scott, Kevin K. Lin, Iain A. McNeish

**Affiliations:** 1 University of Washington, Seattle, WA USA; 2 Clovis Oncology, Inc., Boulder, CO USA; 3 Princess Margaret Cancer Centre, University Health Network, Toronto, ON Canada; 4 BC Cancer—Vancouver, Vancouver, BC Canada; 5 GINECO, Centre Léon Bérard and University Claude Bernard, Lyon, France; 6 Vall d’Hebron University Hospital, Vall d’Hebron Institute of Oncology (VHIO), Barcelona, Spain; 7 The University of Texas, MD Anderson Cancer Center, Houston, TX USA; 8 Memorial Sloan Kettering Cancer Center, New York, NY USA; 9 University of California Los Angeles, Los Angeles, CA USA; 10 The Ohio State University, James Cancer Center, Columbus, OH USA; 11 Gustave Roussy Cancer Center and INSERM U981, Villejuif, France; 12 l’Université de Montréal (CHUM), Montréal, QC Canada; 13 Lawson Health Research Institute, London, ON Canada; 14 University of California San Francisco Helen Diller Family Comprehensive Cancer Center, San Francisco, CA USA; 15 Mayo Clinic, Rochester, MN USA; 16 Rocky Mountain Cancer Centers, Lakewood, CO USA; 17 Tom Baker Cancer Center, Calgary, AB Canada; 18 Guy’s and St. Thomas NHS Foundation Trust, London, UK; 19 Stanford University Cancer Center and Stanford Cancer Institute, Palo Alto, CA USA; 20 University of Melbourne Department of Surgery, Austin Hospital, Heidelberg, VIC Australia; 21 Foundation Medicine, Inc., Cambridge, MA USA; 22 University of Arizona Cancer Center, Tucson, AZ USA; 23 Royal Melbourne Hospital and Walter and Eliza Hall Institute of Medical Research, Parkville, VIC Australia; 24 Imperial College London, London, UK

**Keywords:** Ovarian cancer, Tumour biomarkers

## Abstract

ARIEL2 (NCT01891344) is a single-arm, open-label phase 2 study of the PARP inhibitor (PARPi) rucaparib in relapsed high-grade ovarian carcinoma. In this post hoc exploratory biomarker analysis of pre- and post-platinum ARIEL2 samples, *RAD51C* and *RAD51D* mutations and high-level *BRCA1* promoter methylation predict response to rucaparib, similar to *BRCA1*/*BRCA2* mutations. *BRCA1* methylation loss may be a major cross-resistance mechanism to platinum and PARPi. Genomic scars associated with homologous recombination deficiency are irreversible, persisting even as platinum resistance develops, and therefore are predictive of rucaparib response only in platinum-sensitive disease. The RAS, AKT, and cell cycle pathways may be additional modulators of PARPi sensitivity.

## Abstract (web summary)

The identification of biomarkers of response to PARP inhibitors can enable selection of appropriate ovarian cancer patients for treatment. In this study, the authors report clinical results and exploratory biomarker analyses from the ARIEL2 phase 2 clinical trial on the safety and efficacy of the PARP inhibitor rucaparib in patients with recurrent ovarian cancers

## Introduction

Rucaparib is an inhibitor of poly(ADP-ribose) polymerase (PARP) 1, PARP2, and PARP3, DNA damage repair enzymes in the base excision repair pathway^1^. Homologous recombination deficiency (HRD) sensitizes neoplasms to rucaparib and other DNA-damaging agents (e.g., platinum-based chemotherapy)^2,3^, and platinum sensitivity in high-grade ovarian carcinoma (HGOC) is a strong clinical predictor of benefit from PARP inhibitors (PARPi)^4–7^.

Genetic, epigenetic, and genomic biomarkers can suggest the presence of HRD and help identify patients most likely to respond to PARPi^8–10^. Germline and somatic *BRCA1* or *BRCA2* (*BRCA*) mutations are well-defined biomarkers for PARPi response for a number of cancer types, including breast, ovarian, pancreatic, and prostate^11–16^. Alterations in other homologous recombination repair (HRR) pathway genes, including *PALB2*, *RAD51C*, and *RAD51D*, have been associated with improved responses to rucaparib and other PARPi^17–19^. However, the extent to which many HRR genes contribute to PARPi sensitivity remains unclear^20^. *BRCA1* and *RAD51C* promoter methylation results in transcriptional silencing^21^ and commonly leads to HRD in HGOC^22^. In preclinical studies, *BRCA1* or *RAD51C* methylation led to increased sensitivity to PARPi and platinum^23–25^, but establishing a consistent association of methylation with clinical responses has been elusive^26,27^.

Given the variety of mechanisms that can result in HRD, methods for identifying HRD cancers independent of the mechanisms involved are desirable. HRD cancers exhibit high genomic instability, characterized by deletions of large genomic segments (genome-wide loss of heterozygosity [LOH]), among other genomic aberrations^28,29^. Next-generation sequencing (NGS) can identify multiple patterns of genomic change, including copy number variations, single-nucleotide variations, insertions/deletions, rearrangements characteristic of HRD^29,30^, and others^31–34^. HGOCs with a *BRCA* mutation or *BRCA1/RAD51C* methylation have high levels of genomic LOH^25^. Patients with *BRCA* wild-type (*BRCA*wt) HGOC with high LOH (≥16%) show greater clinical benefit from rucaparib than those with low LOH^7^. High LOH is also correlated with improved response to platinum and other PARPi^6,35^. However, genomic scars accumulate in HRD cancer cells over time and do not disappear when HRR is restored or other PARPi resistance mechanisms develop, making them imperfect biomarkers of PARPi sensitivity^36^. Therefore, understanding the mechanisms leading to HRD and how they might change during prior therapies is important to predict outcomes with PARPi.

ARIEL2 is an international, open-label, 2-part, phase 2 study assessing the safety and efficacy of rucaparib as active treatment in patients with relapsed HGOC. Part 1 enrolled patients with platinum-sensitive disease; clinical results and molecular data from this portion were published^17,25,37,38^. Part 2 enrolled patients who had received three to four prior chemotherapies, including patients with platinum-sensitive or platinum-resistant/refractory disease. Here we present clinical results from Part 2 and post hoc exploratory biomarker analyses using the rich dataset of archival tissue samples and screening biopsies required from patients in both Parts 1 and 2.

## Results

### Part 2 efficacy and safety

Between October 2013 and August 2016, 491 patients were enrolled and received rucaparib in ARIEL2 (Part 1, *n* = 204; Part 2, *n* = 287; Supplementary Fig. S1). Baseline demographics and disease characteristics are presented in Table 1 and Supplementary Table S1. The protocol-prespecified primary endpoints classified patients’ HGOC into one of three HRD groups (*BRCA*-mutant [*BRCA*mut], *BRCA*wt/LOH-high, and *BRCA*wt/LOH-low) using mutations and genome-wide LOH estimates provided by targeted panel NGS of neoplastic tissue (Online Methods)^24^. Among heavily pretreated patients in Part 2, confirmed objective response rates (ORR), the study’s primary endpoint, were 31.0% (95% confidence interval [CI], 21.3–42.0), 6.8% (95% CI, 2.3–15.3), and 5.6% (95% CI, 2.1–11.8), respectively, in patients with *BRCA*mut, *BRCA*wt/LOH-high, and *BRCA*wt/LOH-low HGOC (Supplementary Table S2), with durable responses seen across HRD subgroups (Supplementary Fig. S2a). Data from secondary efficacy endpoints, including ORR by Response Evaluation Criteria In Solid Tumors version 1.1 (RECIST)/Gynecological Cancer InterGroup (GCIG) cancer antigen 125 (CA-125) criteria, progression-free survival (PFS), and overall survival are presented in Supplementary Table S2 and Supplementary Fig. S2b, c.

**Table 1:** Select baseline demographics and disease characteristics.

	ARIEL2 Part 1 (*n* = 204)	ARIEL2 Part 2 (*n* = 287)
Age, median (range), years	64.5 (31.0–86.0)	63.0 (35.0–91.0)
ECOG PS, *n* (%)		
0	134 (65.7)	134 (46.7)
1	70 (34.3)	151 (52.6)
≥2	0	2 (0.7)
Cancer type, *n* (%)		
Epithelial ovarian cancer	164 (80.4)	234 (81.5)
Primary peritoneal cancer	24 (11.8)	28 (9.8)
Fallopian tube cancer	16 (7.8)	25 (8.7)
Histology, *n* (%)^a^		
Serous	197 (96.6)	269 (93.7)
Endometrioid	4 (2.0)	12 (4.2)
Mixed	3 (1.5)	6 (2.1)
No. of prior chemotherapy regimens, median (range)	1 (1–6)	3 (2–5)
No. of prior chemotherapy regimens, *n* (%)		
1	119 (58.3)	0
2	52 (25.5)	2 (0.7)^b^
3	24 (11.8)	186 (64.8)
4	5 (2.5)	97 (33.8)
≥5	4 (2.0)	2 (0.7)
No. of platinum-based regimen, median (range)	1 (1–5)	3 (1–4)
Platinum status, *n* (%)		
Sensitive	202 (99.0)	81 (28.2)
Resistant	2 (1.0)	158 (55.1)
Refractory	0	48 (16.7)
No. of non-platinum regimens following last platinum regimen, *n* (%)		
0	204 (100.0)	81 (28.2)
1	0	150 (52.3)
2	0	52 (18.1)
3	0	4 (1.4)

*ECOG* Eastern Cooperative Oncology Group, *PS* performance status.

^a^Histology based on information provided at enrollment. Pathology re-review triggered by molecular findings reclassified 11 cases as non-high-grade serous or Grade 2/3 endometrioid histology (see Supplementary Table S12).

^b^Receipt of <3 prior chemotherapy regimens was considered a protocol deviation.

The toxicity profile of rucaparib in the heavily pretreated patients in Part 2 (Supplementary Tables S3–S5) was consistent with that previously reported in patients with HGOC^7,37,39^.

### Molecular subgroup profiling across Parts 1 and 2

Taking advantage of the large sample size with varying degree of platinum sensitivity and number of prior treatments, we analyzed the association between molecular characteristics and clinical outcomes across both parts of ARIEL2 to identify molecular mechanisms resulting in rucaparib sensitivity and resistance.

Molecular analyses were used to characterize HGOC based on mutation, methylation, and molecular subgroup status. In these post hoc analyses, molecular subgroups were determined using a 16% LOH cutoff for LOH-high in the *BRCA*wt subgroup; this refined cutoff was validated within ARIEL2 Part 1 (ref. ^40^) and was prospectively tested in the maintenance setting in ARIEL3 (NCT01968213)^7^. To define the *BRCA*mut subgroup comprehensively, we utilized both available local testing data and an updated *BRCA* missense mutation classifier to reclassify the HGOC from 14 patients as *BRCA*mut, beyond those initially identified by targeted NGS (Supplementary Fig. S3, Online Methods). In total, 138 HGOC were classified as *BRCA*mut, 156 were *BRCA*wt/LOH-high, 168 were *BRCA*wt/LOH-low, and 29 were *BRCA*wt/LOH-unclassified.

### Efficacy based on molecular subgroup and platinum status

**Fig. 1:**
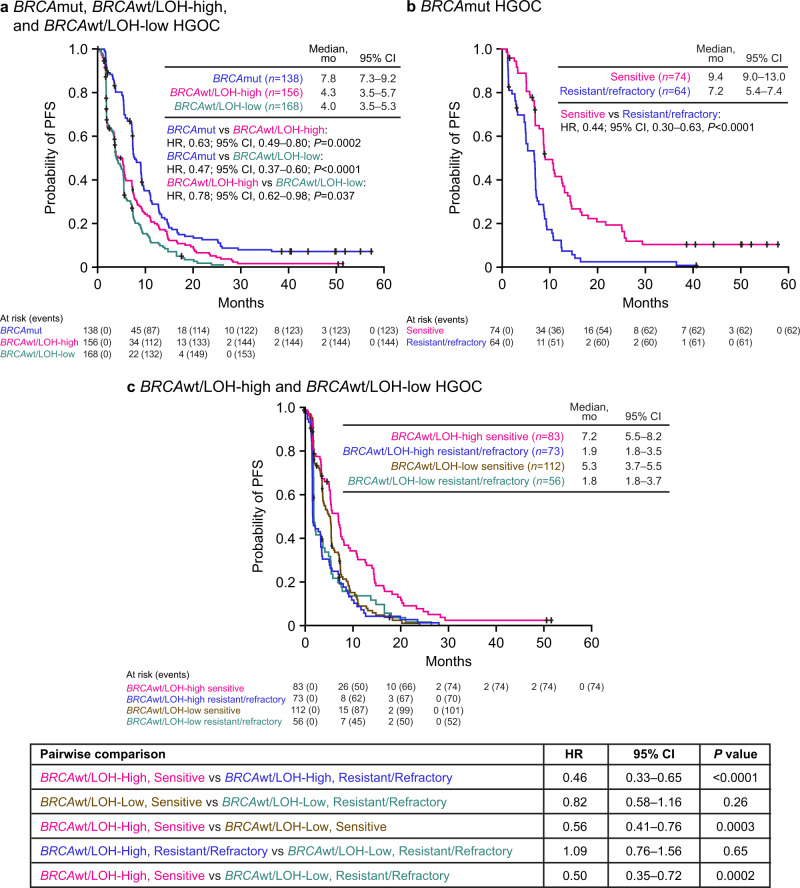
PFS by molecular subgroup and platinum-sensitivity status. **a** PFS in patients with *BRCA*mut (blue), *BRCA*wt/LOH-high (magenta), and *BRCA*wt/LOH-low (teal) HGOC. **b** PFS in patients with *BRCA*mut HGOC that are platinum resistant/refractory (blue) or platinum sensitive (magenta). **c** PFS in patients with *BRCA*wt/LOH-high HGOC that are platinum resistant/refractory (blue) or platinum sensitive (magenta) and PFS in patients with *BRCA*wt/LOH-low HGOC that are platinum resistant/refractory (teal) or platinum sensitive (brown). *P* values were computed using a Cox proportional hazard model. The interaction between molecular subgroup and platinum status was also tested in the Cox proportional hazard model and found to be significant (*P* < 0.05). *BRCA* *BRCA1* or *BRCA2*, CI confidence interval, HGOC high-grade ovarian carcinoma, HR hazard ratio, LOH loss of heterozygosity, mut mutated, PFS progression-free survival, wt wild type.

**Fig. 2:**
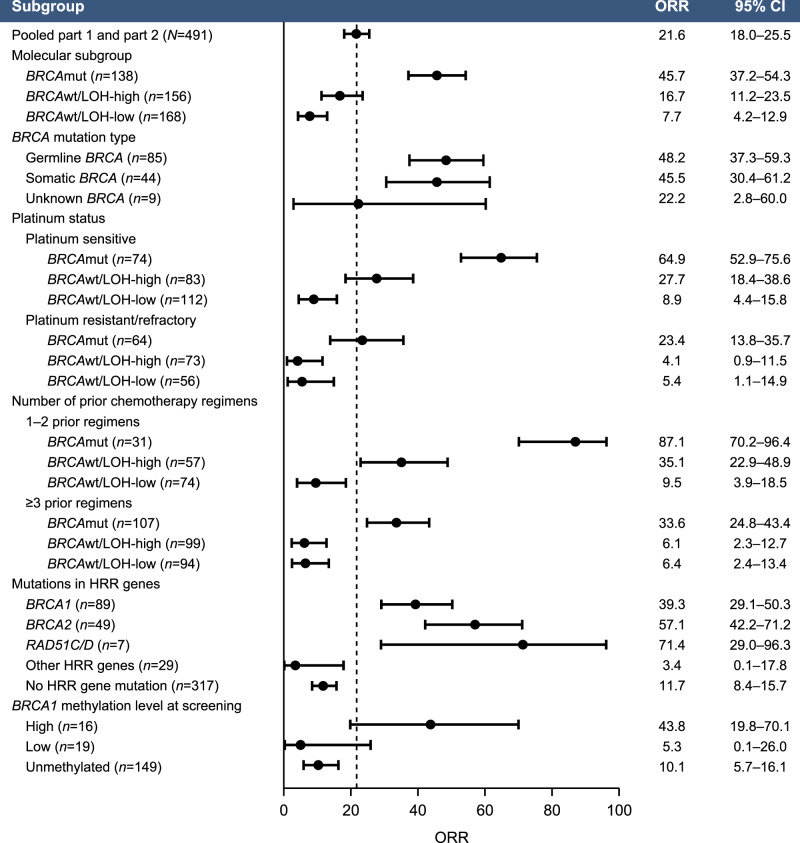
ORR based on post hoc molecular subgroups and baseline clinical characteristics. Data are plotted as ORR (dots) with the corresponding two-sided 95% CI (error bars) based on the Clopper–Pearson method. *BRCA* *BRCA1* or *BRCA2*, CI confidence interval, HRR homologous recombination repair, LOH loss of heterozygosity, mut mutated, ORR objective response rate, wt wild type.

PFS and ORR differed significantly among molecular subgroups. Patients with *BRCA*mut HGOC had superior outcomes, with a median PFS of 7.8 months and ORR of 45.7% (95% CI, 37.2–54.3). In contrast, among patients with *BRCA*wt/LOH-high and *BRCA*wt/LOH-low HGOC, median PFS was 4.3 months and 4.0 months and ORR was 16.7% (95% CI, 11.2–23.5) and 7.7% (95% CI, 4.2–12.9), respectively (Figs. 1a and 2 and Supplementary Table S6).

We hypothesized that cross-resistance mechanisms that emerge during prior lines of platinum treatment affect rucaparib sensitivity. Among patients with *BRCA*mut HGOC, those who were sensitive to the most recent line of platinum therapy (platinum-free interval [PFI] >6 months) had a median PFS of 9.4 months and ORR of 64.9% (95% CI, 52.9–75.6), whereas those with platinum-resistant (PFI <6 months) or platinum-refractory (progression on prior platinum) disease performed significantly worse, with a median PFS of 7.2 months (hazard ratio [HR], 0.44; 95% CI, 0.30–0.63; *P* < 0.0001) and ORR of 23.4% (95% CI, 13.8–35.7; *P* < 0.0001, Fisher’s exact test) (Figs. 1b and 2 and Supplementary Table S6). Among patients with *BRCA*wt HGOC, the LOH-high, platinum-sensitive subgroup performed better (median PFS, 7.2 months; ORR, 27.7% [95% CI, 18.4–38.6]) than the LOH-high, platinum-resistant/refractory subgroup (median PFS, 1.9 months [HR, 0.46; 95% CI, 0.33–0.65]; ORR, 4.1% [95% CI, 0.9–11.5]) and the LOH-low subgroups regardless of platinum status (Fig. 1c). Genomic scarring is, therefore, a good predictor of rucaparib sensitivity before the emergence of cross-resistance, while LOH-low HGOC lacks PARPi-sensitizing HRD mechanisms, and therefore shows little rucaparib efficacy even with limited prior platinum exposure. Indeed, rucaparib efficacy in *BRCA*mut and *BRCA*wt/LOH-high but not *BRCA*wt/LOH-low HGOC was better for patients with platinum-sensitive disease who had received one or two prior therapies (Part 1) than those who had three or four prior chemotherapy regimens with predominantly platinum-resistant/refractory disease (Part 2) (Fig. 2 and Supplementary Table S6). These observations suggest that platinum sensitivity and fewer lines of prior platinum treatments are both linked to better outcomes on rucaparib in HRD-associated HGOC; this finding is further supported by multivariate analyses that identified platinum status and number of prior chemotherapy regimens as significant predictors for ORR (Supplementary Tables S7 and S8). Other patient demographics and baseline disease characteristics (age, body mass index, race, Eastern Cooperative Oncology Group performance status, type of ovarian cancer) also tested in multivariate analysis were not identified as significant predictors for response.

### HRD-associated genetic events leading to rucaparib sensitivity

In view of the association between *BRCA* mutation status and PARPi response^7,37^, we examined the relationship between rucaparib response and HRR gene mutation status. Deleterious germline or somatic *BRCA* mutations were detected in HGOC from 28% of patients (138/491; *n* = 89 *BRCA1*, *n* = 49 *BRCA2*). Consistent with prior analyses of rucaparib treatment in patients with BRCAmut HGOC, similar ORRs were observed between patients with HGOC associated with germline or somatic *BRCA* mutations (48.2% [95% CI, 37.3–59.3] and 45.5% [30.4–61.2]; Fig. 2). All somatic *BRCA* mutations were present at similar variant allele fractions to concurrent *TP53* mutations, consistent with a driver mutation, and no subclonal somatic *BRCA* mutations were identified. *BRCA* reversion mutations restoring the open reading frame were enriched in patients with platinum-resistant/refractory disease and were associated with poor responses to rucaparib^38^. None of the 10 patients who had a reversion mutation before initiating rucaparib achieved a confirmed response (6 progressive disease [PD]; 4 stable disease [SD]). Reversion mutations accounted for 6/16 (37.5%) of *BRCA*mut HGOC whose best response on rucaparib was PD.

We examined whether alterations in 28 HRR genes beyond *BRCA* (Supplementary Table S9) correlated with rucaparib sensitivity. Patients with *BRCA*wt HGOC associated with any deleterious HRR gene mutation (36/491; 7.3%) had a median PFS of 5.7 months and ORR of 16.7% (95% CI, 6.4–32.8), which was not different from that of patients with *BRCA*wt HGOC not associated with an HRR gene mutation (median PFS, 3.7 months [HR, 0.83; 95% CI, 0.58–1.20; *P* = 0.32]; ORR, 11.7% [95% CI, 8.4–15.7]; Supplementary Fig. S4a, Supplementary Table S10 and Fig. 2).

Given strong evidence linking mutations in *PALB2*, *RAD51C*, and *RAD51D* to PARPi sensitivity^17,37,41^, we examined separately how HGOC harboring mutations in any of these three genes responded. Seven patients had HGOC with a deleterious *RAD51C/D* mutation (*n* = 4 *RAD51C*, *n* = 3 *RAD51D*), and none had a *PALB2* mutation. The response rate among the seven patients with *RAD51C/D*-mutated HGOC was high (5/7; 71.4%; 95% CI, 29.0–96.3; Fig. 2). With the exception of one responder with an *NBN* mutation (Supplementary Table S10), all responders with HGOC harboring a non-*BRCA* HRR gene mutations had a *RAD51C* or a *RAD51D* alteration; results from the multivariate analysis also identified *RAD51C/D* mutation as a significant predictor of ORR (Supplementary Tables S7 and S8). Median PFS among patients with *RAD51C/D*-mutated HGOC was similar to that of patients with *BRCA*mut HGOC (11.0 and 7.8 months, respectively; HR, 1.52; 95% CI, 0.67–3.44; *P* = 0.32; Supplementary Fig. S4b). Although 6/7 *RAD51C/D*-mutated HGOC were platinum sensitive, one patient had platinum-resistant disease and achieved a partial response to rucaparib, with a PFS of 13.0 months. Additionally, 4/7 patients with *RAD51C*/*D-*mutated HGOC had received three or more lines of prior chemotherapy regimens, suggesting that as long as cross-resistance is not present, rucaparib can be highly effective in HGOC with *RAD51C/D* mutations, even in late lines of treatment.

### HRD-associated epigenetic events leading to rucaparib sensitivity

We assessed the presence of both *BRCA1* and *RAD51C* promoter methylation by methylation-specific polymerase chain reaction (MSP)^25^ in available biopsies obtained from archival HGOC tissues (*n* = 321), and at screening prior to initiating rucaparib (*n* = 230). Consistent with published estimates (11–15% for *BRCA1* and 1–3% for *RAD51C*)^22,42^, ARIEL2 methylation frequencies were 16.8% for *BRCA1* and 1.6% for *RAD51C* in archival tissues, and 13.5% for *BRCA1* and 2.6% for *RAD51C* at screening. None of the *BRCA*mut HGOC included in the MSP analysis (*n* = 79) exhibited *BRCA1* promoter methylation, suggesting that *BRCA* mutations and *BRCA1* promoter methylation are mutually exclusive HRD mechanisms.

Interestingly, we saw no difference in PFS based on either archival or screening methylation status in patients with *BRCA*wt HGOC, suggesting that the mere presence of methylation at the two promoters is not a biomarker for rucaparib outcomes (Supplementary Fig. S5).

MSP analysis detects the presence of methylation but does not estimate what fraction of the neoplastic cells is methylated or if all alleles are silenced^25^. To determine the fraction of methylated *BRCA1* and *RAD51C* copies, we employed methylation-sensitive digital droplet polymerase chain reaction (MS-ddPCR). Initial results from Part 1 platinum-sensitive samples analyzed for quantitative *BRCA1* methylation have been published^25^. Here, we report MS-ddPCR analysis of all ARIEL2 patients with *BRCA1* or *RAD51C* promoter methylation detectable by MSP in at least one time point or for whom MSP data were inconclusive. Of the 99 samples submitted (56 archival; 43 screening), 82 were identified as methylated or unmethylated by both methods; four samples that were unmethylated by MSP showed very low levels of methylation by MS-ddPCR (all <2%), suggesting that their methylation levels were below the level of detection of MSP. Methylation in two samples was detected by MSP but was not confirmed by MS-ddPCR. The remaining samples analyzed by MS-ddPCR did not have MSP data available. Only two *RAD51C* methylated cases, as determined by MSP, were available for MS-ddPCR analysis. Quantitative analysis confirmed low (<70%) *RAD51C* methylation in the screening and archival samples for both; however, due to this small sample size, *RAD51C* methylated cases were excluded from quantitative methylation analysis.

Using a cutoff of 70% to define high methylation^25^, we observed lower *BRCA1* RNA expression among samples with high *BRCA1* methylation compared with unmethylated samples (*P* < 0.0001, Wilcoxon rank-sum test) (Supplementary Fig. S6a). However, samples with low methylation had intermediate *BRCA1* expression similar to unmethylated cases (*P* = 0.079, Wilcoxon rank-sum test). This analysis is consistent with *BRCA1* promoter methylation repressing gene expression, with high and low methylation resulting in different levels of *BRCA1* suppression and, therefore, differential impact on HRR activity.

**Fig. 3:**
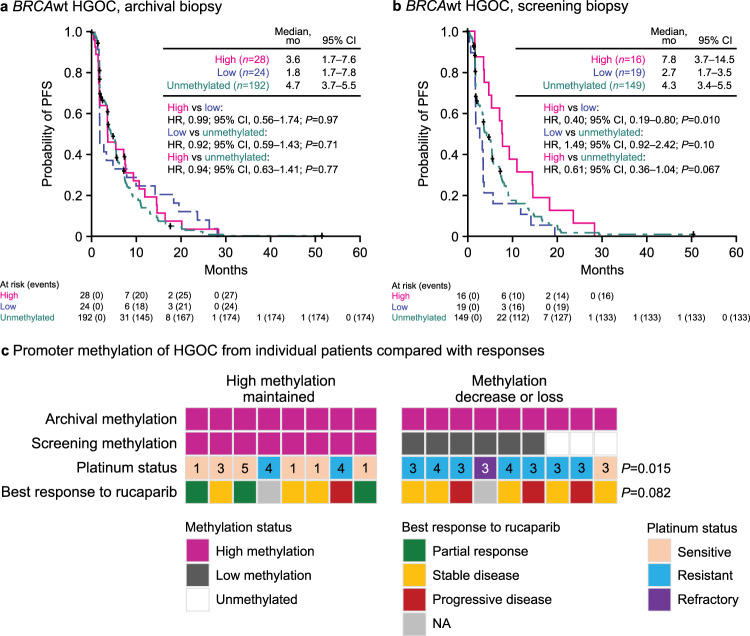
High *BRCA1* methylation levels at screening are associated with better outcomes. **a** Kaplan–Meier plot showing PFS in ARIEL2 patients with *BRCA*wt HGOC having high (magenta), low (blue), or no methylation (unmethylated, teal) in archival biopsy. **b** Kaplan–Meier plot showing PFS in ARIEL2 patients with *BRCA*wt HGOC having high (magenta), low (blue), or no methylation (unmethylated, green) in screening biopsy. *P* values in panels **a** and **b** are based on Cox proportional hazard model. **c** Methylation status at screening as compared to the archival sample, platinum status, and best response to rucaparib of 17 HGOC with high *BRCA1* methylation levels in the archival sample and an available matched screening biopsy; numbers indicate number prior lines of chemotherapy treatment; *P* values are based on a two-sided Fisher’s exact test testing the proportion of patients with platinum status of sensitive and best response of partial response between HGOC that maintained methylation vs decrease or loss of methylation. *BRCA* *BRCA1* or *BRCA2*, CI confidence interval, HGOC high-grade ovarian carcinoma, HR hazard ratio, NA not applicable, PFS progression-free survival, wt wild type.

Although archival *BRCA1* methylation levels were not associated with differential PFS with rucaparib treatment (Fig. 3a), PFS was better in patients with *BRCA*wt HGOC containing high *BRCA1* methylation immediately prior to initiating rucaparib than those with unmethylated or low levels of methylation (Fig. 3b). Median PFS in patients with high methylation HGOC immediately prior to initiating rucaparib was similar to that of patients with *BRCA*mut HGOC without reversion mutations (7.8 vs 9.0 months; HR, 0.84; 95% CI, 0.50–1.42; *P* = 0.52), whereas patients with HGOC with low methylation had a similar median PFS as patients with HGOC with *BRCA* reversion mutations (2.7 vs 1.8 months; HR, 0.73; 95% CI, 0.33–1.62; *P* = 0.44) (Supplementary Fig. S6b). The ORR was 43.8% (95% CI, 19.8–70.1) in patients with high methylation HGOC, 5.3% (95% CI, 0.1–26.0) in patients with low methylation HGOC, and 10.1% (95% CI, 5.7–16.1) in patients with unmethylated HGOC (Fig. 2). This difference in response rates indicates that decreased *BRCA1* methylation may be an acquired resistance mechanism leading to lower rucaparib efficacy, similar to *BRCA* reversion mutations. Consistent with these findings, methylation status was identified as a significant predictor of ORR in addition to *BRCA* and *RAD51C/D* mutation, in a multivariate analysis (Supplemental Tables S7 and S8).

To examine whether a decrease in methylation correlates with resistance to prior therapies and poor response to rucaparib, we focused on patients with *BRCA*wt HGOC whose archival samples showed high methylation and who had a matched screening biopsy prior to rucaparib treatment (*n* = 17) (Fig. 3c). HGOCs that maintained high methylation across archival and screening biopsies were enriched for platinum-sensitive disease (6/8), while those with a decrease or loss in methylation were predominantly platinum resistant/refractory (8/9) (*P* = 0.015, Fisher’s exact test). Loss/decrease of methylation was also associated with higher number (≥3) of prior lines of chemotherapy treatment (*P* = 0.029, Fisher’s exact test; Fig. 3c).

The ORR among the eight patients with HGOC that maintained high methylation was 38%, whereas no responses were observed among the nine with HGOC with methylation decrease or loss (Fig. 3c), indicating that demethylation may be associated with reduced rucaparib efficacy (*P* = 0.082, Fisher’s exact test). Although based on a small number of cases, this analysis suggests that methylation plasticity is an important factor in developing cross-resistance to both platinum agents and PARPi via de-repression of *BRCA1* expression and reactivation of HRR. *BRCA1* methylation, both high and low, is highly enriched in *BRCA*wt/LOH-high HGOC compared with other molecular subgroups (Supplementary Table S11; *P* < 0.0001, Chi-square test), confirming an association between methylation and accumulation of genome scars^43^. In ARIEL2, 14% of *BRCA*wt/LOH-high HGOC (13/94) had high *BRCA1* methylation at screening, and 18% (17/94) had low *BRCA1* methylation. Outcomes in the two groups were vastly different with ORRs of 46.2% (95% CI, 19.2–74.9) and 5.9% (95% CI, 0.1–28.7; *P* = 0.025, Fisher’s exact test) in patients with *BRCA*wt/LOH-high HGOC with high vs low screening methylation, respectively, and median PFS of 9.3 vs 3.3 months (HR, 0.44; 95% CI, 0.21–0.92; *P* = 0.015). Thus, acquired resistance by demethylation before initiating rucaparib may account for a substantial fraction of PARPi resistance among *BRCA*wt/LOH-high HGOC, a population expected to be PARPi responsive.

### Non-HRR gene alterations may modulate response to rucaparib

**Fig. 4:**
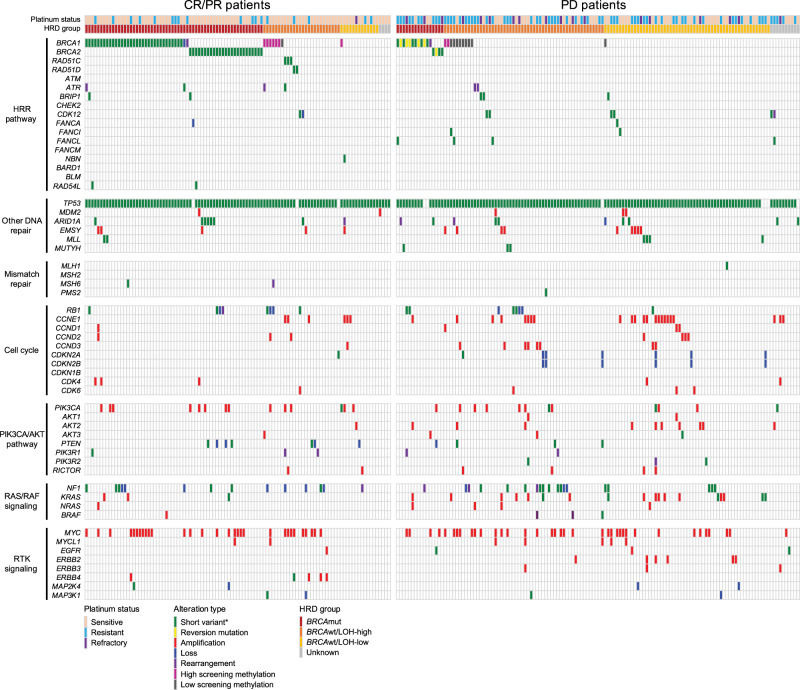
Genetic and epigenetic alteration landscape of HGOC with confirmed best response of CR/PR (left) or PD (right). Methylation levels shown are at screening. *Short variant include nonsense, missense, frameshift, and splice site alterations. All reported alterations are deleterious or likely deleterious. *BRCA* *BRCA1* or *BRCA2*, CR complete response, HGOC high-grade ovarian carcinoma, HRR homologous recombination repair, LOH loss of heterozygosity, mut mutated, PD progressive disease, PR partial response, RTK receptor tyrosine kinase, wt wild type.

Apart from the HRR pathway, alterations in other pathways may also affect rucaparib response. Mutation and copy number data for 315 genes based on targeted carcinoma NGS were available for 484 of 491 patients. The genetic and epigenetic alterations of HGOC from patients who achieved a confirmed complete or partial response (*n* = 103) and those whose best response was PD (*n* = 136) are shown in Fig. 4; patients who achieved a best response of SD are summarized in Supplementary Fig. S7. Genes and pathways commonly altered in ovarian cancer were included. *TP53* was the most frequently mutated gene (92.8%), consistent with published data^22,44^. We also observed frequent alterations in genes involved in non-HRR DNA repair, cell cycle regulation, PI3K/AKT signaling, RAS pathway, and receptor tyrosine kinase signaling.

Although events in most non-HRR genes are individually rare and did not show significantly different alteration rates between responders and nonresponders, a few interesting observations can be noted. *CCNE1* amplification was mutually exclusive with both *BRCA* mutations, as previously reported^22,45^, and *BRCA1* methylation. *KRAS* and *NRAS* amplifications were enriched among *BRCA*wt HGOC with PD (14/120, 11.7%); in contrast, no amplification was identified in *BRCA*wt responders (0/43, *P* = 0.023, Fisher’s exact test), suggesting that dysregulated RAS signaling may lead to poorer outcomes in *BRCA*wt HGOC.

Several patients (*n* = 14) had a *KRAS*, *NRAS*, or *BRAF* activating mutation in the absence of a *TP53* mutation, a genetic profile associated with low-grade serous carcinoma, mucinous carcinoma, and mesonephric-like adenocarcinoma^46–48^. A blinded pathology re-review confirmed the presence of non-high-grade serous or non-grade 2/3 endometrioid histologies in 11 of the 14 cases, while all cancers with *KRAS*, *NRAS*, or *BRAF* mutations and a *TP53* alteration (*n* = 7) were confirmed to be high-grade serous (Supplementary Table S12 and Supplementary Fig. S8). Eleven of 12 cancers with *KRAS*, *NRAS*, or *BRAF* activating mutations in *TP53* wild-type background that could be classified into molecular subgroups were LOH-low, consistent with the observation that low grade and mesonephric-like histologies are not driven by HRD^49^. Although none of the 14 patients achieved a clinical response to rucaparib, indicating that PARPi are not likely to be active in these rare histologies, some experienced PFS longer than 200 days, an observation that may be indicative of slower overall tumor growth^47,50^.

Amplification and overexpression in *CCNE1*/cell cycle and AKT pathway genes have been previously associated with platinum resistance^42,51^. Among platinum-resistant/refractory patients in ARIEL2, patients with *BRCA*wt HGOC and alterations (predominantly via copy number change) either in the *AKT1/2/3* genes or cell cycle pathway at screening had poorer PFS outcomes compared with other patients with *BRCA*wt HGOC (AKT: HR, 0.45; 95% CI, 0.24–0.85; *P* = 0.013; cell cycle: HR, 0.66; 95% CI, 0.44–1.00; *P* = 0.050; Supplementary Fig. S9), consistent with the hypothesis that certain platinum resistance mechanisms may modulate responses to other DNA-damaging agents, including PARPi.

## Discussion

ARIEL2 assessed the safety and efficacy of rucaparib in an unselected HGOC population. Platinum sensitivity was a strong clinical predictor of rucaparib response in this PARPi-naïve population, especially in the *BRCA*mut and *BRCA*wt/LOH-high molecular subgroups. As HRD is a common mechanism leading to both platinum and PARPi sensitivity^1^, this finding suggests that cross-resistance mechanisms arising during platinum therapies also impact responses to PARPi. Here, we describe HRD mechanisms leading to both platinum and rucaparib sensitivity (*BRCA* mutation, *RAD51C/D* alterations, and high *BRCA1* promoter methylation) and summarize two important cross-resistance mechanisms: *BRCA* reversion mutations, which we have previously shown^38^, and loss of *BRCA1* methylation described here for the first time using archival and screening clinical specimens. Other cross-resistance mechanisms to PARPi have been previously proposed, including *BRCA1* alternative splicing^52^, *53BP1* loss^53,54^, increased expression of *BRCA* hypomorphs^55–57^, and *ABCB1* gene fusions^42,58^. These mechanisms may also play a role in ARIEL2 but were not assessed here due to insufficient patient samples.

Alterations in *RAD51C* and *RAD51D* correlated with meaningful clinical activity of rucaparib similar to that of *BRCA*mut HGOC. Therefore, we propose utilizing panels incorporating *RAD51C/D* when considering targeted therapies. Importantly, we previously showed that reversion mutations also occur in *RAD51C/D* as a resistance mechanism^17^, supporting their essential role in generating synthetic lethality with PARPi.

The effect of mutations in other HRR genes on rucaparib sensitivity remains unclear given the low relative frequency of each gene in the ARIEL2 cohort. Although it has been hypothesized that *ATM* mutations are correlated with sensitivity to PARP inhibition, in a recent phase 3 trial, there was no survival benefit seen for patients with prostate cancer associated with *ATM* mutations who were treated with olaparib vs androgen-receptor targeted therapy (HR, 0.93; 95% CI, 0.53–1.75)^59^. In ARIEL2, no responses were observed among five patients with HGOC harboring an *ATM* mutation. However, three of these patients had platinum-resistant/refractory disease, and only one of the remaining two was evaluable for response. Therefore, with the limited data, the impact of *ATM* on rucaparib sensitivity in HGOC remains inconclusive.

**Fig. 5:**
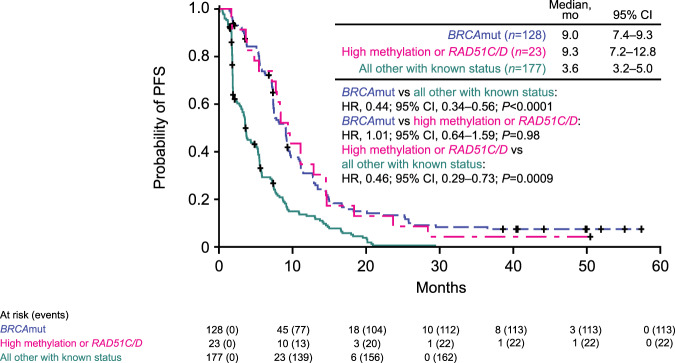
High *BRCA1* methylation at pretreatment or the presence of a *RAD51C/D* mutation result in similar PFS as the presence of *BRCA* mutations. PFS of patients with *BRCA*mut HGOCs, excluding cases with reversion mutations (blue), patients with HGOCs harboring a *RAD51C/D* mutation or high methylation at pretreatment (magenta), and all other patients with known mutation and methylation status, including cases with reversion mutations (teal). *P* values were computed using a Cox proportional hazard model. BRCA *BRCA1* or *BRCA2*, CI confidence interval, HGOC high-grade ovarian carcinoma, HR hazard ratio, mut mutated, PFS progression-free survival.

Through quantitative MS-ddPCR assessment of *BRCA1* methylation in archival and screening biopsies, we show that high-level methylation of the *BRCA1* promoter is a strong biomarker of rucaparib sensitivity. Patients with carcinomas harboring this modification or a *RAD51C/D* mutation had PFS similar to that of patients with *BRCA*mut HGOC treated with rucaparib, even after cases with *BRCA* reversion mutations are removed from comparison (Fig. 5). Further supporting this finding, each of these characteristics (methylation status, *BRCA* mutation, or *RAD51C/D* mutation) were identified as a significant predictor for ORR in multivariate analysis. Given strong preclinical data linking *BRCA1* promoter methylation to HRD, and platinum and PARPi sensitivity^23,24^, the previous lack of definitive clinical evidence associating *BRCA1* methylation and therapy response has been perplexing^60,61^. The TNT trial (NCT00532727) found no association between *BRCA1* methylation and response to carboplatin vs docetaxel in patients with triple-negative breast cancer^26^. Discrepancies between these findings and our own may have both biological and technical explanations. First, most studies analyze methylation in samples obtained at diagnosis, thus missing methylation loss that may have occurred since sample collection. Second, previous studies only report the binary presence or absence of *BRCA1* promoter methylation without considering the levels of methylation observed. All *BRCA1* alleles likely need to be methylated to establish complete gene silencing and subsequent HRD^26^. Importantly, we show that methylation should be quantitatively assessed immediately prior to treatment to ensure high enough levels to result in sufficient promoter silencing, especially in later-line settings.

Relying on genomic scarring as evidence of HRD in later lines of treatment poses significant disadvantages. Once established, genomic scars persist and do not provide a real-time predictor of sensitivity after multiple treatment lines. In the platinum-sensitive ARIEL2 Part 1 cases, high LOH was associated with a higher ORR among *BRCA*wt HGOC. But among the more heavily pretreated Part 2 patients, high LOH did not predict rucaparib sensitivity. Notably, all carcinomas with acquired platinum resistance (i.e., *BRCA* reversion mutation and *BRCA1* promoter demethylation) remained LOH-high. Thus, accumulation of genomic scarring is an irreversible process, persisting even as cancers re-acquire functional HRR. In the QUADRA trial of the PARPi niraparib after three lines of therapy, which used the myChoice HRD test to determine HRD status, *BRCA*mut HGOC were included in the HRD group, driving much of the 15% ORR^62^. When only considering the *BRCA*wt cases in QUADRA, HRD was associated with a higher ORR only for platinum-sensitive disease (20% vs 4% for non-HRD) and was not predictive for platinum-resistant cases (2.4% vs 3% non-HRD), similar to our findings. Therefore, although stratifying *BRCA*wt HGOC for PARPi treatment based on evidence of accumulated genomic scarring is useful early in the disease course, these tests lose utility in platinum-resistant/refractory cases.

We also examined alterations in non-HRR pathways that may indirectly modulate response to rucaparib. Cell cycle gene alterations, including amplifications of cyclin E, cyclin D, and *CDK4/6* genes and deletions in the cell cycle inhibitors *CDKN2A/B* and *CDKN1B*, were common in *BRCA*wt HGOC in ARIEL2 and have been associated with platinum resistance^22,63^. AKT signaling has also been linked to platinum resistance, and AKT inhibition appears to reverse resistance in certain models^64^. In ARIEL2, platinum-resistant/refractory *BRCA*wt HGOC with alterations in the cell cycle pathway and the *AKT1/2/3* genes showed poorer outcomes on rucaparib, implying that these pathways may be relevant to responses to multiple DNA-damaging agents. Further in vitro and in vivo studies would be essential to confirm these clinical observations, describe the mechanistic connections between these pathways and PARPi, and determine if combination therapies with PARPi and cell cycle or AKT inhibitors may be beneficial for certain late-stage HGOC patients. Most of the cancers with *KRAS*, *NRAS*, or *BRAF* activating mutations in the absence of a *TP53* mutation were subsequently found to be non-HGOC, and the lack of response to rucaparib in these cancers indicates the importance of confirming histological subtype when these mutations are detected.

Overall, our analysis highlights significant overlap between molecular mechanisms resulting in platinum and PARPi sensitivity and the extent of cross-resistance that exists between these two drug classes. In addition to *BRCA* mutations, *RAD51C*/*D* mutations and high-level *BRCA1* promoter methylation are strong predictors of sensitivity to a PARPi. Given its general tolerability and efficacy, especially in patients with *BRCA*mut and *BRCA*wt/LOH-high HGOC, and data supporting the use of PARPi as maintenance following primary therapy^8–10,65^, we propose that administration of rucaparib, as active treatment, should be considered in earlier lines of therapy, before the emergence of platinum resistance. Such an approach would increase the likelihood of patients experiencing significant clinical benefit while maintaining the improved quality of life associated with targeted vs systemic therapy.

## Methods

### Study design and participants

ARIEL2 (CO-338-017; NCT01891344) was an international, multicenter, two-part, phase 2 open-label study conducted across 64 sites in Australia, Canada, France, Spain, the United Kingdom, and the United States. The study design and clinical results from Part 1 were published previously^37^. In ARIEL2, eligible patients were aged 18 years or older with histologically confirmed, relapsed, high-grade serous or Grade 2 or Grade 3 endometroid epithelial ovarian, fallopian tube, or primary peritoneal cancer. Patients were required to have measurable disease according to RECIST, an Eastern Cooperative Oncology Group Performance Status of 0 or 1, and adequate organ function. Part 1 enrolled patients with relapsed HGOC who had received at least one prior platinum-based regimen and had platinum-sensitive disease (disease progression ≥6 months after last platinum). Part 2 enrolled patients with relapsed HGOC who had received three to four prior chemotherapies and had a treatment-free interval of more than 6 months following first-line chemotherapy. Patients in Part 2 could be platinum sensitive, platinum resistant (disease progression <6 months after last platinum, with best response other than PD), or platinum refractory (best response of PD on last platinum with progression-free interval <2 months). Both Part 1 and Part 2 enrolled patients regardless of their HRD status, with the exception that Part 1 had a cap on the number of patients with a known germline *BRCA* mutation (*n* = 15) allowed, in order to enrich for the *BRCA*wt population.

Patients were ineligible if they previously received a PARPi in either the treatment setting or as a maintenance therapy, had an active second malignancy, had central nervous system metastases, or received anticancer therapy 14 days or fewer before receiving their first dose of rucaparib.

The study was approved by national or local institutional review boards, as appropriate at each site (see Supplementary Information for full list), and was carried out in accordance with the Declaration of Helsinki and Good Clinical Practice Guidelines of the International Conference on Harmonization. Patients provided written informed consent before participation.

### Procedures

Patients were treated with oral rucaparib at 600 mg twice daily until disease progression, unacceptable toxicity, or death. Supportive care (e.g., antiemetics or analgesics for pain control) was permitted at the investigator’s discretion. Treatment interruptions and dose reductions (in decrements of 120 mg formulation in Part 1 and 100 mg in Part 2) were permitted if a patient had a grade 3 or greater adverse event. Treatment was discontinued if a dose interruption occurred for more than 14 consecutive days (longer dose interruptions were permitted with sponsor approval).

Tumor response was assessed by the investigators using RECIST, with computed tomography scans at screening and every 8 weeks (±4 days) during treatment and after treatment for patients who discontinued for any reason other than disease progression. Patients who had been on study at least 18 months could have the frequency of disease assessment decreased to every 16 weeks (±2 days). Assessment continued until confirmed disease progression, death, start of subsequent treatment, or loss to follow-up. Serum CA-125 measures were taken at screening, day 1 of each cycle, and the end of treatment, or when clinically indicated. Hematology, serum chemistry, and safety assessments were done at screening, day 1 and day 15 of cycle 1, and day 1 of any subsequent cycles. Adverse events were classified in accordance with the Medical Dictionary for Drug Regulatory Activities classification system version 19.1 and graded for severity in accordance with the National Cancer Institute’s Common Terminology Criteria for Adverse Event version 4.03 (https://nciterms.nci.nih.gov/ncitbrowser/start.jsf). Part 1 patients were treated and followed up until disease progression, death, or discontinuation of treatment due to other reasons. Part 2 patients were followed for survival, subsequent therapy, and secondary malignancy every 12 weeks until death, loss to follow-up, withdrawal of consent from study, or study closure, whichever happened first.

### Molecular subgroup classification

Biomarker analysis was performed following the REMARK (Reporting Recommendations for Tumor Marker Prognostic Studies) guidelines^66^. A known HRR gene mutation was not required for enrollment in ARIEL2. Patients were required to have adequate formalin-fixed paraffin-embedded (FFPE) archival tumor tissue available and to have undergone a pretreatment (screening) biopsy (optional in patients with HGOC having a known deleterious germline *BRCA* mutation in Part 2). For the ARIEL2 Part 1 and Part 2 prespecified analyses, tumor *BRCA* status was determined centrally using the FoundationOne^TM^ NGS assay (Supplementary Fig. S3)^37,67^.

Local *BRCA* test results were collected when available (Supplementary Fig. S3). For our post hoc analysis of the pooled ARIEL2 patient population, we classified a patient as *BRCA*mut if a deleterious *BRCA* alteration was detected by either central tumor testing or local testing (Supplementary Fig. S3). The majority of *BRCA* alterations that were detected by local testing but not by tissue testing were large rearrangements, known to be challenging to detect by NGS.

FoundationOne^TM^ was also used to calculate the percentage of genomic LOH in archival and screening biopsies and to identify alterations in genes other than *BRCA*^37,67^.

To classify *BRCA*wt patients into LOH-high and LOH-low subgroups, we used a prespecified cutoff of 14% for patients in Part 1 and a prespecified cutoff of 18% for patients in Part 2 (Supplementary Fig. S3). For the post hoc, exploratory molecular subgroup analyses, both the local and central test information for *BRCA* was utilized. For *BRCA*wt, we utilized an optimized cutoff of 16%, which was shown to be the optimal cutoff in ARIEL2 Part 1 (ref. ^40^) and was prospectively evaluated in the phase 3 ARIEL3 study (Supplementary Fig. S3)^7^.

*BRCA1* and *BRCA2* germline/somatic status was determined by a combination of methods. Blood samples from all ARIEL2 Part 1 *BRCA*mut samples and a subset of ARIEL2 Part 2 *BRCA*mut cases (*n* = 74) were analyzed using the BROCA homologous recombination sequencing assay (University of Washington, Seattle WA, USA). Any alteration detected by FoundationOne™ NGS but not BROCA was considered somatic. The remainder of the samples were assigned germline/somatic status based on local testing data provided by the study sites (*n* = 29) or computational inference method using the targeted NGS data (*n* = 26)^68^. Computational inference was not attempted for samples with tumor purity >80%; those samples were listed as Unknown. Nine *BRCA*mut cases remained with unknown germline/somatic status. Agreement between germline/somatic status determined through germline sequencing (BROCA and local testing) and the computational inference method was very high for samples that had both data available (*n* = 67, 95.5% agreement, kappa = 0.88, *P* < 0.0001, Cohen’s Kappa statistics).

HRR gene mutation subgroup was based on alterations in the genes listed in Supplementary Table S6. Germline/somatic status for ARIEL2 Part 1 non-*BRCA* HRR genes was determined by the BROCA homologous recombination sequencing assay (University of Washington, Seattle, WA, USA). Any alteration detected by FoundationOne™ NGS but not BROCA was considered somatic. *BRCA*wt ARIEL2 Part 2 samples were not analyzed by central germline testing and germline/somatic status for these cases was determined by computational inference as described above^68^. The same computational approach was used to determine zygosity for all non-*BRCA* HRR gene alterations^68^.

The detection of *BRCA* reversion mutations has been described in detail previously^38^. In short, for reversion mutations present in cell-free DNA (cfDNA), plasma samples collected from ARIEL2 patients were analyzed using the Guardant360 (ref. ^69^) or FoundationACT^70^ cfDNA assays. Central tumor tissue NGS data were also re-analyzed to determine if reversion mutations were present. *BRCA* reversion mutations were defined as: (i) a base substitution that changed a nonsense mutation to a missense mutation, (ii) an insertion/deletion that restored the ORF, or (iii) a larger intragenic deletion that deleted the primary deleterious mutation. Reversion mutations were detected in 10/112 (8.9%) *BRCA*mut HGOC pretreatment samples: nine by Guardant360 plasma analysis (eight previously described^38^ and one identified in post-publication analysis) and one by central tumor tissue NGS analysis.

Patients were classified as having a cell cycle pathway alteration if an alteration in any of the following genes was detected in their screening cancer sample: *CCNE1*, *CCND1*, *CCND2*, *CCND3*, *CDKN2A*, *CDKN2B*, *CDKN1B*, *CDK4*, or *CDK6*. Patients were classified as having an *AKT* alteration if they had an alteration in *AKT1*, *AKT2*, or *AKT3* in their screening cancer sample. Amplifications reported were high-copy gains (CN > 5).

### Methylation analysis

The presence of methylation at the *BRCA1* and *RAD51C* promoters was detected using MSP, as previously described^37^. In summary five 10 μm sections of FFPE tissue were deparaffinized, rehydrated, and digested with Proteinase K (Zymo Research, Irvine, CA, USA) overnight and bisulfite conversion of 10 μL of supernatant was performed in duplicates using the EZ DNA Methylation-Direct kit (Zymo Research). Following bisulfite conversion, the samples underwent desulfonation and cleanup, and 2 μL of bisulfite-converted DNA was evaluated with MSP for *BRCA1* and *RAD51C* using primers listed in Supplementary Table S13.

Quantification of *BRCA1* and *RAD51C* methylation levels in HGOC from ARIEL2 Part 1 and Part 2 patients was performed by quantitative MS-ddPCR methodology as previously described^25^: DNA extracted from FFPE-preserved tissue sections was bisulfite converted using the EZ DNA Methylation-Lightning kit (Zymo Research). *BRCA1* primers were designed for a 72 bp amplicon in the untranslated region (UTR) (Supplementary Table S13). *RAD51C* methylation, primers were designed for a 142 bp amplicon in the *RAD51C* UTR. Minor groove binder probes hybridizing to the fully unmethylated (2′-chloro-7′phenyl-1,4-dichloro-6-carboxyfluorescein [VIC] labeled) and the fully methylated sequences (6-carboxyfluorescein [FAM] labeled) were used. The ddPCR was performed on the Bio-Rad QX-200 system. Methylation frequencies determined by MS-ddPCR were normalized to neoplastic purity and *BRCA1/RAD51C* copy number estimates using the following formula: RM × (CN × TP + (100 − TP) × 2)/(TP × CN)/100, where RM is the raw fraction methylated copies detected, TP is the tumor purity, and CN is the *BRCA1* copy number^25^. The tumor purity and copy number were estimated based on the targeted NGS data^68^. Patient samples were classified as having high or low methylation levels based on a predefined cutoff of 70%^25^. In the absence of contradicting MS-ddPCR data, biopsies with 0% *BRCA1* promoter methylation by MS-ddPCR, or ones determined to be unmethylated by MSP, were labeled as unmethylated.

### *BRCA1* expression analysis

For a subset of the samples analyzed for *BRCA1* promoter methylation status, we assessed the *BRCA1* RNA expression levels by NanoString^71^.

### Pathology analysis and histology reclassification

For all patients, hematoxylin and eosin (H&E) staining was performed following standard procedures on archival and/or screening biopsy tissue corresponding to the tissue that was submitted for central NGS testing. The H&E slides for the patients harboring activating mutations in the *KRAS*/*NRAS*/*BRAF* genes were re-reviewed by gynecologic pathologists (J.A.E. and D.I.L.), and a consensus diagnosis was rendered. At time of re-review, the pathologists were blinded to the genomic findings for these patients. Both archival and screening tissue H&E slides were available for at least two patients each for mesonephric-like carcinomas, low-grade serous carcinomas, and endometrioid adenocarcinomas, and the reclassification was confirmed in both samples. Representative fields were selected to describe features concidered for reclassification.

### Statistical analysis

All analyses were performed using the safety population, which included all patients who received at least one dose of rucaparib. The primary endpoint in Part 1 was PFS by predefined HRD subgroups. PFS was defined as the number of days from the first dose of study drug to disease progression by RECIST, as determined by the investigator, or death due to any cause, whichever occurred first. Patients without a documented event of progression were censored on the date of their last adequate cancer assessment (i.e., radiologic assessment) or the date of the first dose of the study drug if no cancer assessments were performed. In Part 2, the primary endpoint was ORR, defined as the proportion of patients achieving a best response of complete or partial response according to RECIST as assessed by the investigator by predefined HRD subgroups. Secondary endpoints included the proportion of patients achieving an objective response (according to RECIST and GCIG CA-125 criteria), duration of response (according to RECIST), and overall survival. Response endpoints were summarized with frequencies and percentage using Clopper–Pearson methodology to calculate 95% CIs. Rates of response were compared with pair-wise comparison using Fisher’s exact test. The response (partial or complete response) by RECIST needed to be confirmed by a second assessment after at least 4 weeks. Duration of confirmed response (complete or partial response) was calculated from the initial date a response was detected to the first date of PD. Patients without a documented event of progression were censored on the date of their last adequate cancer assessment (i.e., radiologic assessment) or date of response if no cancer assessments were performed. Overall survival was defined as the number of days from the date of first dose of study drug to the date of death (due to any cause). Patients without a known date of death were censored on the date the patient was last known to be alive. Duration of response, PFS, and overall survival were summarized with Kaplan–Meier methodology, including median estimates and 95% CIs using log–log distribution. In addition, a Cox proportional hazard model was used to summarize these endpoints and make comparisons between molecular subgroups. Here, we also present post hoc exploratory molecular biomarker analyses of PFS and ORR outcomes in order to further explore clinical benefit. Most of the post hoc analyses are based on univariate comparisons. In addition, a stepwise multivariate logistics regression model was used to identify predictors of confirmed response (PR or CR) using baseline and molecular characteristic variables as predictors in the model (Supplementary Tables S7 and S8). For all time-to-event analyses, the proportional hazards assumption was assessed visually with log–log plots (Supplementary Fig. S10); as the assumption of the model was valid in each instance, we provide HR and 95% CI for these endpoints. All *P* values for the post hoc exploratory analyses of molecular subgroups are presented for descriptive purposes only.

Comparisons of proportions between methylation subgroups were summarized using Fisher’s exact test or chi-square tests; for the analysis of number prior lines of chemotherapy treatment, patients were grouped in 1–2 and ≥3 prior lines subgroups. The distribution of *BRCA1* gene expression levels was analyzed using a nonparametric Wilcoxon rank-sum test to compare molecular subgroups. Agreement analyses were performed using non-weighted Cohen’s Kappa statistics.

Data analysis was performed using SAS v9.4 (SAS Institute, Cary, NC, USA) and Excel 2016 (Microsoft Corporation, Redmond, WA, USA).

### Reporting summary

Further information on research design is available in the Nature Research Reporting Summary linked to this article.

## Supplementary information

Supplementary Information

Reporting Summary

## Data Availability

Consent was not obtained from patients to allow posting of the data to public repositories. Requests for de-identified datasets for the results reported in this publication will be made available to qualified researchers following submission of a methodologically sound proposal to medinfo@clovisoncology.com. Data will be made available for such requests following online publication of this article and for 1 year thereafter in compliance with applicable privacy laws, data protection, and requirements for consent and anonymization. Data will be provided by Clovis Oncology. The redacted protocol for the ARIEL2 clinical study is available on thelancet.com: https://ars.els-cdn.com/content/image/1-s2.0-S1470204516305599-mmc1.pdf. Clovis Oncology does not share identified participant data or a data dictionary.
