# Supplementary material for: Molecular and clinical determinants of response and resistance to rucaparib for recurrent ovarian cancer treatment in ARIEL2 (Parts 1 and 2)
Source: Nat Commun. 2021 May 3;12:2487. doi: 10.1038/s41467-021-22582-6 (PMC8093258; doi:10.1038/s41467-021-22582-6)
Supplement: Supplementary file 1 — Supplementary Information [file 41467_2021_22582_MOESM1_ESM.pdf]

Supplementary Information

Ethics approval

The study was approved by by the following institutional review boards and ethics committees for participating centers: Alberta Cancer Research Ethics Committee (ACREC), BC Cancer Agency - Vancouver Cancer Centre, CEIm Hospital Clínico Universitario de Valencia, Comité d'Éthique de la recherche du CHUM, Dana-Faber Cancer Institute Office For Human Research Studies, Fox Chase Cancer Center IRB, France CPP No., Fred Hutchinson Cancer Research Center Institutional Review Office, Health Research Ethics Board of Alberta Cancer Committee, Jewish General Hospital Research Ethics committee, Johns Hopkins Institutional Review Board, Massachusetts General Hospital Partners Human Research Committee, Mayo Clinic IRB, Melbourne Health HREC, Melbourne Health Office For Research, Memorial Sloan Kettering Cancer Center IRB/Privacy Board, Mercy Health HREC, Mission Health System IRB, Northern Sydney Local Health District Research Governance Office, NYU School of Medicine Institutional Review Board, Ontario Cancer Research Ethics Board, Orlando Regional Medical Center IRB, Quorum Review IRB (Now Advarra), Royal Brisbane and Women's Hospital Service District, Human Research Ethics Committee, Sir Charles Gairdner and Osborne Park Health Care Group, Human Research Ethics Committee, South Eastern Sydney Local Health District, Southern Adelaide Clinical Human Research Ethics Committee, Stanford University Institutional Review Board, The University of Texas MD Anderson Cancer Center IRB, Texas Oncology Austin Brain Tumor Center, UBC BC Cancer Agency Research Ethics Board, UCLA Office of Human Research Protection Program, UC San Diego Human Research Protections Program, University of California San Francisco Human Research Protection Program, University of Miami Human Subject Research Office, University of Oklahoma Health Sciences Center IRB, University of Pennsylvania IRB, Washington University in St. Louis Human Research Protection Office, West of Scotland Research Ethics Service, Western Institutional Review Boards, Western Sydney Local Health District Research Governance Office.

Supplementary Table S1 | Additional baseline demographics and disease characteristics

	**ARIEL2 Part 1 (n=204)**	**ARIEL2 Part 2 (n=287)**
Optimally debulked, n (%)		
Yes	156 (76.5%)	227 (79.1%)
No	36 (17.6%)	40 (13.9%)
Not known	12 (5.9%)	20 (7.0%)
Most common platinum-resistant agents[a], n (%)		
Liposomal doxorubicin monotherapy	0	72 (34.0)
Bevacizumab + chemotherapy (paclitaxel, PLD or topotecan)	0	29 (13.7)
Paclitaxel monotherapy	0	25 (11.8)
Gemcitabine monotherapy	0	19 (9.0)
Topotecan monotherapy	0	12 (5.7)
Liposomal doxorubicin + trabectedin	0	8 (3.8)
Other	0	47 (22.2)

[a]Categories are not mutually exclusive
PLD, pegylated liposomal doxorubicin

Supplementary Table S2 | Primary and secondary endpoint by prespecified HRD subgroup in ARIEL2 Part 2

	Primary endpoint confirmed objective response by RECIST, n/N (%) [95% CI]	Secondary endpoint of confirmed objective response rate by combined RECIST and CA-125 response, n/N (%) [95% CI]
BRCA mutant	26/84 (31.0) [21.3–42.0]	46/84 (54.8) [43.5–65.7]
BRCA wild type and LOH high[a]	5/73 (6.8) [2.3–15.3]	9/73 (12.3) [5.8–22.1]
BRCA wild type and LOH low	6/107 (5.6) [2.1–11.8]	14/107 (13.1) [7.3–21.0]
BRCA wild type and LOH unclassified[b]	3/23 (13.0) [2.8–33.6]	7/23 (30.4) [13.2–52.9]

[a]For LOH high, a cutoff of ≥18% genomic LOH was used. [b]Tumor sample was not evaluable for percentage of genomic LOH due to low tumor content or low aneuploidy.
BRCA, *BRCA1* or *BRCA2*; CA-125, cancer antigen 125; CI, confidence interval; HRD, homologous recombination deficiency; LOH, loss of heterozygosity; RECIST, Response Evaluation Criteria in Solid Tumors version 1.1.

Supplementary Table S3 | Summary of safety in ARIEL2 Part 2

	**ARIEL2 Part 2 (N=287)**
Treatment duration, median (range), months	3.5 (0.1–46.7)
At least 1 TEAE, n (%)	287 (100)
At least 1 TEAE grade ≥3, n (%)	186 (64.8)
At least 1 serious TEAE, n (%)	92 (32.1)
Treatment interruption and/or dose reduction due to TEAE, n (%)	186 (64.8)
Treatment interruption due to TEAE	172 (59.9)
Dose reduction due to TEAE	134 (46.7)
Discontinued due to a TEAE, n (%)	69 (24.0)
Death due to a TEAE, n (%)	18 (6.3)
Malignant neoplasm progression	10 (3.5)
Nonprogression AE leading to death	8 (2.8)

AE, adverse event; TEAE, treatment-emergent adverse event.

Supplementary Table S4 | Most common (≥10% of patients) TEAEs of any grade in ARIEL2 Part 2

TEAE	ARIEL2 Part 2 (N=287)	
	**Any grade, n (%)**	**Grade ≥3, n (%)**
Nausea	219 (76.3)	16 (5.6)
Asthenia or fatigue	212 (73.9)	33 (11.5)
Anemia or decreased hemoglobin	127 (44.3)	63 (22.0)
Vomiting	126 (43.9)	17 (5.9)
Decreased appetite	112 (39.0)	11 (3.8)
ALT or AST increased	101 (35.2)	26 (9.1)
Abdominal pain	93 (32.4)	12 (4.2)
Dysgeusia	93 (32.4)	1 (0.3)
Constipation	84 (29.3)	5 (1.7)
Diarrhea	84 (29.3)	5 (1.7)
Thrombocytopenia or decreased platelets	78 (27.2)	25 (8.7)
Dyspnea	68 (23.7)	3 (1.0)
Blood creatinine increased	65 (22.6)	2 (0.7)
Edema peripheral	38 (13.2)	1 (0.3)
Abdominal pain (upper)	36 (12.5)	3 (1.0)
Urinary tract infection	36 (12.5)	8 (2.8)
Dizziness	35 (12.2)	0
Headache	35 (12.2)	0
Pyrexia	34 (11.8)	0
Abdominal distension	32 (11.1)	0
Cough	30 (10.5)	0
Insomnia	30 (10.5)	0
Neutropenia or decreased ANC	30 (10.5)	17 (5.9)
Weight decreased	30 (10.5)	1 (0.3)
Hypomagnesemia	29 (10.1)	2 (0.7)

ALT, alanine aminotransferase; ANC, absolute neutrophil count; AST, aspartate aminotransferase; TEAE, treatment-emergent adverse event.

Supplementary Table S5 | MDS/AML incidence on treatment and after treatment discontinuation

	MDS/AML incidence, n (%)	
	**On treatment[a]**	**After treatment discontinuation**
**ARIEL2 Part 1 (n=204)**	**0**	**1 (0.5)**
BRCA mutant	0	0
BRCA wild type/LOH high	0	1 (0.5)
**ARIEL2 Part 2 (n=287)**	**2 (0.7)**	**3 (1.0)**
BRCA mutant	1 (0.3)	2 (0.7)
BRCA wild type/LOH high	1 (0.3)	1 (0.3)

[a]Occurring while on rucaparib treatment or during the 28-day safety follow-up.
AML, acute myeloid leukemia; BRCA, *BRCA1* or *BRCA2;* LOH, loss of heterozygosity; MDS, myelodysplastic syndrome.

Supplementary Table S6 | ORR by molecular subgroups and prior lines of therapy, and platinum status

	% (n/N) [95% CI]														
	***BRCA*mut**<br>**(n=138)**			***BRCA*wt/LOH-high[a]**<br>**(n=156)**			***BRCA*wt/LOH-low**<br>**(n=168)**			***BRCA*wt/LOH unclassified[b]**<br>**(n=29)**			**Overall**<br>**(n=491)**		
	**Prior Chemotherapy Regimens**														
	**All**	**1–2**	**≥3**	**All**	**1–2**	**≥3**	**All**	**1–2**	**≥3**	**All**	**1–2**	**≥3**	**All**	**1–2**	**≥3**
**Platinum Status**															
**Sensitive (n=283)**	**64.9**<br>**(48/74)**<br>[52.9–75.6]	**87.1**<br>**(27/31)**<br>[70.2–96.4]	**48.8**<br>**(21/43)**<br>[33.3–64.5]	**27.7**<br>**(23/83)**<br>[18.4–38.6]	**35.7**<br>**(20/56)**<br>[23.4–49.6]	**11.1**<br>**(3/27)**<br>[2.4–29.2]	**8.9**<br>**(10/112)**<br>[4.4–15.8]	**9.6**<br>**(7/73)**<br>[3.9–18.8]	**7.7**<br>**(3/39)**<br>[1.6–20.9]	**28.6**<br>**(4/14)**<br>[8.4–58.1]	**30.0**<br>**(3/10)**<br>[6.7–65.2]	**25.0**<br>**(1/4)**<br>[0.6–80.6]	**30.0**<br>**(85/283)**<br>[24.8–35.7]	**33.5**<br>**(57/170)**<br>[26.5–41.2]	**24.8**<br>**(28/113)**<br>[17.1–33.8]
**Resistant (n=160)**	**30.0**<br>**(15/50)**<br>[17.9–44.6]		**30.0**<br>**(15/50)**<br>[17.9–44.6]	**5.5**<br>**(3/55)**<br>[1.1–15.1]	**0**<br>**(0/1)**<br>[NA, NA]	**5.6**<br>**(3/54)**<br>[1.2–15.4]	**4.7**<br>**(2/43)**<br>[0.6–15.8]	**0**<br>**(0/1)**<br>[NA, NA]	**4.8**<br>**(2/42)**<br>[0.6–16.2]	**0**<br>**(0/12)**<br>[0.0–26.5]	**0**<br>**(0/1)**<br>[NA, NA]	**0**<br>**(0/11)**<br>[0.0–28.5]	**12.5**<br>**(20/160)**<br>[7.8–18.6]	**0**<br>**(0/3)**<br>[0.0–70.8]	**12.7**<br>**(20/157)**<br>[8.0–19.0]
**Refractory (n=48)**	**0**<br>**(0/14)**<br>[0.0–23.2]		**0**<br>**(0/14)**<br>[0.0–23.2]	**0**<br>**(0/18)**<br>[0.0–18.5]		**0**<br>**(0/18)**<br>[0.0–18.5]	**7.7**<br>**(1/13)**<br>[0.2–36.0]		**7.7**<br>**(1/13)**<br>[0.2–36.0]	**0**<br>**(0/3)**<br>[0.0–70.8]		**0**<br>**(0/3)**<br>[0.0–70.8]	**2.1**<br>**(1/48)**<br>[0.1–11.1]		**2.1**<br>**(1/48)**<br>[0.1–11.1]
**Total (n=491)**	**45.7**<br>**(63/138)**<br>[37.2–54.3]	**87.1**<br>**(27/31)**<br>[70.2–96.4]	**33.6**<br>**(36/107)**<br>[24.8–43.4]	**16.7**<br>**(26/156)**<br>[11.2–23.5]	**35.1**<br>**(20/57)**<br>[22.9–48.9]	**6.1**<br>**(6/99)**<br>[2.3–12.7]	**7.7**<br>**(13/168)**<br>[4.2–12.9]	**9.5**<br>**(7/74)**<br>[3.9–18.5]	**6.4**<br>**(6/94)**<br>[2.4–13.4]	**13.8**<br>**(4/29)**<br>[3.9–31.7]	**27.3**<br>**(3/11)**<br>[6.0–61.0]	**5.6**<br>**(1/18)**<br>[0.1–27.3]	**21.6**<br>**(106/491)**<br>[18.0–25.5]	**32.9**<br>**(57/173)**<br>[26.0–40.5]	**15.4**<br>**(49/318)**<br>[11.6–19.9]

Grey cells indicate ORR ≥10%.
[a]For LOH high, a cutoff of ≥16% genomic LOH was used.
[b]HGOC sample was not evaluable for percentage of genomic LOH due to low neoplastic content or low aneuploidy.
BRCA, *BRCA1* or *BRCA2*; CI, confidence interval; HGOC, high-grade ovarian carcinoma; LOH, loss of heterozygosity; mut, mutated; NA, not available; ORR, objective response rate; wt, wild type.

Supplementary Table S7 | Multivariate logistic regression model for ORR. A stepwise multivariate logistics regression model was used to identify predictors of confirmed response (partial response or complete response) including the following baseline characteristics (n=491): age, body mass index, race, Eastern Cooperative Oncology Group performance status, type of ovarian cancer, number of prior chemotherapy regimens, platinum status (sensitive, resistant, refractory), and genomic characteristics.

The number of prior chemotherapy regimens (*P*=0.0011), platinum status (*P*=0.0061), and the genomic characteristics (*P*<0.0001) were found to be significant predictors in the model using a two-sided significance level of 0.05. All other baseline characteristics had *P* values >0.2 and not included in the model.

**Parameter**	**Estimate**	**Standard error**	***P* value**
Intercept	−0.9381	0.6118	0.1252
Number of prior chemotherapy regimens	−0.5210	0.1593	0.0011
Platinum status			
Resistant	0.1224	0.4074	0.7638
Sensitive	1.1387	0.4006	0.0045
Genomic characteristics			
BRCA mutation	1.7191	0.3661	<0.0001
RAD51C/D mutation	1.8552	1.0391	0.0742
Other HRR gene mutation	−1.8893	0.9244	0.0410
HRRwt – methyl high	1.1984	0.5688	0.0351
HRRwt – methyl low	−0.7958	0.9717	0.4128
HRRwt – methyl NA	−0.9147	0.3845	0.0173

Reference categories that are an exact linear combinations of the other categories for corresponding predictor is not displayed (eg, platinum status of refractory and genomic characteristics of HRRwt unmethylated).
BRCA, *BRCA1* or *BRCA2*; HRR, homologous recombination repair; methyl, methylation; NA, not available; wt, wild type.

Supplementary Table S8 | Odds ratio estimates for the significant predictors of response. Consistent with the findings of the univariate analyses of these subgroups, the multivariate logistrics regression model identified known prognostics factors such as number of prior chemotherapy regimens and platinum status, as well as HRR gene and methylation as significant predictors for confirmed response in the patient population (n=491).

**Effect**	**Odds ratio**	**95% CI**
Number of prior chemotherapy regimens	0.594	0.435–0.812
Platinum status		
Resistant vs Refractory	3.989	0.477–33.391
Sensitive vs Refractory	11.022	1.337–90.888
Genomic characteristics		
BRCA mutation vs HRRwt unmethylated	18.030	7.575–42.917
RAD51C/D mutation vs HRRwt unmethylated	20.658	1.865–228.889
Other HRR gene mutation vs HRRwt unmethylated	0.489	0.057–4.152
HRRwt – methyl high vs unmethylated	10.711	2.852–40.230
HRRwt – methyl low vs unmethyldated	1.458	0.153–13.878
HRRwt – methyl NA vs unmethyldated	1.295	0.541–3.100

BRCA, *BRCA1* or *BRCA2*; CI, confidence interval; HRR, homologous recombination repair; methyl, methylation; NA, not available; wt, wild type.

Supplementary Table S9 | List of HRR genes defined by protocol

BRCA	**non-*BRCA* HRR**	
BRCA1	*ATM*	*FANCL*
BRCA2	*ATR*	*FANCM*
	ATRX	*MRE11A*
	BARD1	*NBN*
	BLM	*PALB2*
	BRIP1	*RAD50*
	CHEK1	*RAD51*
	CHEK2	*RAD51B*
	FANCA	*RAD51C*
	FANCC	*RAD51D*
	FANCD2	*RAD52*
	FANCE	*RAD54L*
	FANCF	*RPA1*
	FANCG	
	FANCI	

HRR, homologous recombination repair.

Supplementary Table S10 | Characteristics of patients with non-*BRCA* HRR gene mutations

Patient #	Study Part	Gene	Protein effect	Zygosity	Germline/ Somatic status	Molecular subgroup	Platinum Status	No. prior chemo. regimens	PFS (mo)	Best response on rucaparib
1	Part 1	**ATM**	homozygous deletion	NA	Somatic	NA	Resistant	1	5.1	SD
2	Part 2	**ATM**	R1618*	heterozygous	Somatic[a]	*BRCA*wt/LOH-low	Refractory	3	21.2	SD
3	Part 2	**ATM**	splice site 4437-1G>T	NA	NA	NA	Resistant	3	18.2	SD
4	Part 2	**ATM**<br>CHEK2	R2443*<br>T367fs*15	homozygous<br>heterozygous	Germline[a]<br>Germline[a]	*BRCA*wt/LOH-low	Sensitive	3	7.0	SD
5	Part 1	**ATM**	G2644fs*2	homozygous	Somatic	*BRCA*wt/LOH-high	Sensitive	1	0.0+	NE
6	Part 2	**BLM**	Y736fs*5	NA	Germline[a]	NA	Resistant	4	3.9	SD
7	Part 2	**BRIP1**	W1002*	homozygous	NA	*BRCA*wt/LOH-high	Resistant	4	10.1	SD
8	Part 2	**BRIP1**	N97fs*3	homozygous	Germline[a]	*BRCA*wt/LOH-high	Refractory	3	2.0	PD
9	Part 2	**BRIP1**	E458*	homozygous	Germline[a]	*BRCA*wt/LOH-high	Sensitive	3	1.9	PD
10	Part 1	**BRIP1**	splice site 93+1G>T	heterozygous	Germline[a]	*BRCA*wt/LOH-low	Sensitive	1	5.5	SD
11	Part 1	**BRIP1**	K752fs*12	homozygous	Germline	*BRCA*wt/LOH-low	Sensitive	1	2.5+	SD
12	Part 2	**BRIP1**	R798*	homozygous	Germline[a]	*BRCA*wt/LOH-low	Sensitive	4	1.2	PD
13	Part 1	**CHEK2**	splice site 1008_1008+1GG>TT	homozygous	Somatic	*BRCA*wt/LOH-low	Sensitive	1	7.2	SD
14	Part 1	**CHEK2**	Q83fs*27	heterozygous	Germline	*BRCA*wt/LOH-high	Sensitive	3	5.6	SD
15	Part 2	**CHEK2**	T367fs*15	NA	Germline[a]	*BRCA*wt/LOH-low	Sensitive	4	5.7	SD
16	Part 2	**FANCA**	Q1437*	homozygous	Germline[a]	*BRCA*wt/LOH-low	Resistant	3	0.4	PD

Patient #	Study Part	Gene	Protein effect	Zygosity	Germline/ Somatic status	Molecular subgroup	Platinum Status	No. prior chemo. regimens	PFS (mo)	Best response on rucaparib
17	Part 2	**FANCI**	L1294fs*10	NA	NA	*BRCA*wt/LOH-high	Resistant	3	1.8	PD
18	Part 1	**FANCI**	I466fs*7	heterozygous	Germline	*BRCA*wt/LOH-low	Sensitive	2	1.7	PD
19	Part 2	**FANCL**	T367fs*12+	heterozygous	Germline[a]	*BRCA*wt/LOH-low	Sensitive	3	11.1	SD
20	Part 2	**FANCL**	T367fs*12+	heterozygous	Germline[a]	*BRCA*wt/LOH-high	Resistant	4	1.8	PD
21	Part 2	**FANCL**	T367fs*12+	heterozygous	Germline[a]	NA	Resistant	4	0.7	PD
22	Part 2	**FANCL**	T367fs*12+	heterozygous	Germline[a]	*BRCA*wt/LOH-high	Resistant	4	1.8	PD
23	Part 2	**FANCM**	K863fs*12	NA	NA	*BRCA*wt/LOH-low	Sensitive	3	3.6	SD
24	Part 2	**FANCM**	K117*	homozygous	NA	*BRCA*wt/LOH-low	Sensitive	3	0.7	NE
25	Part 1	**NBN**	K233fs*5	heterozygous	Germline	NA	Sensitive	1	5.4	SD
26	Part 1	**NBN**	K219fs*16	heterozygous	Germline	*BRCA*wt/LOH-low	Sensitive	1	10.5	**PR**
27	Part 1	**RAD51B**	R47*	heterozygous	Germline	*BRCA*wt/LOH-low	Sensitive	2	7.5	SD
28	Part 1	**RAD51C**	R193*	homozygous	Germline	*BRCA*wt/LOH-high	Sensitive	1	9.7	**PR**
29	Part 1	**RAD51C**<br>ATR	R370*<br>D1572fs*1	NA<br>heterozygous	Germline<br>Somatic	*BRCA*wt/LOH-high	Sensitive	1	51.1+	**PR**
30	Part 1	**RAD51C**	splice	homozygous	Germline	*BRCA*wt/LOH-high	Sensitive	3	8.5	SD
31	Part 1	**RAD51C**	splice site 572-2A>G	homozygous	Germline	*BRCA*wt/LOH-high	Sensitive	3	8.4	**PR**
32	Part 1	**RAD51D**	G146fs*50	homozygous	Germline	*BRCA*wt/LOH-high	Sensitive	2	11.2	SD
33	Part 2	**RAD51D**	R120*	homozygous	Germline[a]	*BRCA*wt/LOH-high	Resistant	4	13.0	**PR**

Patient #	Study Part	Gene	Protein effect	Zygosity	Germline/ Somatic status	Molecular subgroup	Platinum Status	No. prior chemo. regimens	PFS (mo)	Best response on rucaparib
34	Part 2	**RAD51D**	Q79*	homozygous	Germline[a]	*BRCA*wt/LOH-high	Sensitive	4	14.7	**PR**
35	Part 2	**RAD54L**	splice site 1610+1G>A	heterozygous	Somatic[a]	*BRCA*wt/LOH-low	Resistant	3	5.5	SD
36	Part 1	**RAD54L**	F591fs*1	NA	Somatic	*BRCA*wt/LOH-low	Sensitive	2	7.4	SD

Bolded gene names indicate primary/driving mutation.
[a]Germline/somatic status determined using computational inference method.
+, patient was censored; BRCA, *BRCA1* or *BRCA2*; HRR, homologous recombination repair; LOH, loss of heterozygosity; NA, not available; NE, not evaluable; PD, progressive disease; PFS, progression-free survival; PR, partial response; SD, stable disease; wt, wild type.

Supplementary Table S11 | Number of cases with high, low, or no (unmethylated) *BRCA1* promoter methylation at screening in evaluated *BRCA*mut, LOH-high, and LOH-low HGOC

**Molecular subgroup, n (%)**	**High *BRCA1* methylation (n=16)**	**Low *BRCA1* methylation (n=19)**	**Unmethylated (n=193)**
*BRCA*mut	0	0	44 (22.8)
*BRCA*wt/LOH-high	13 (81.3)	17 (89.5)	62 (32.1)
*BRCA*wt/LOH-low	2 (12.5)	1 (5.3)	79 (40.9)
*BRCA*wt/LOH unknown	1 (6.3)	1 (5.3)	8 (4.1)

*BRCA*wt/LOH-high HGOC are significantly enriched for the presence of high and low methylation of the *BRCA1* promoters (P<0.0001, Chi-square test).

BRCA, *BRCA1* or *BRCA2*; HGOC, high-grade ovarian carcinoma; LOH, loss of heterozygosity; mut, mutated; wt, wild type.

Supplementary Table S12 | Retrospective blinded pathologist review of ovarian carcinoma with *KRAS*/*NRAS*/*BRAF* mutations

Patient #	RAS/BRAF mutation	TP53 Status	Molecular subgroup	Platinum status	PFI (months)	Best response	PFS (days)	Local pathology diagnosis		Pathology re-review with integrated genomic analysis	Evidence of histology other than HGSC or G2/3 endometrioid
1	KRAS G12V	wt	*BRCA*wt/LOH-low	Resistant	5.6	PD	51	ENDOMETRIOID	HIGH GRADE	Mesonephric-like carcinoma	Yes
2	BRAF V600E	wt	*BRCA*wt/LOH-low	Refractory	0.4	SD	635	SEROUS	HIGH GRADE	LGSC	Yes
3	KRAS G12V	wt	Unknown	Resistant	5.5	SD	547	ENDOMETRIOID	HIGH GRADE	NA	NA
4	NRAS Q61R	wt	*BRCA*wt/LOH-low	Sensitive	9.2	SD	166	SEROUS	HIGH GRADE	LGSC	Yes
5	KRAS G12D	wt	*BRCA*wt/LOH-low	Sensitive	12.1	SD	226	SEROUS	HIGH GRADE	High-grade carcinoma with serous features	No
6	NRAS Q61R	wt	*BRCA*wt/LOH-low	Sensitive	50.8	SD	142	SEROUS	HIGH GRADE	LGSC	Yes
7	BRAF G469A[a]	wt	*BRCA*wt/LOH-high	Resistant	5.8	PD	72	ENDOMETRIOID	HIGH GRADE	G2 endometrioid adenocarcinoma	No
8	KRAS G12V	wt	Unknown	Resistant	5.8	SD	109	SEROUS	HIGH GRADE	LGSC	Yes
9	KRAS G12D	wt	*BRCA*wt/LOH-low	Resistant	2.2	SD	229	ENDOMETRIOID	HIGH GRADE	Mesonephric-like carcinoma	Yes
10	KRAS G12D	wt	*BRCA*wt/LOH-low	Sensitive	9.4	SD	211	MIXED	HIGH GRADE	G1 endometrioid adenocarcinoma	Yes
11	KRAS G12D	wt	*BRCA*wt/LOH-low	Resistant	2.8	SD	277	ENDOMETRIOID	HIGH GRADE	Mesonephric-like carcinoma	Yes
12	KRAS Q61H, BRAF G466V	wt	*BRCA*wt/LOH-low	Sensitive	71.5	SD	57	ENDOMETRIOID	HIGH GRADE	G1 endometrioid adenocarcinoma	Yes
13	KRAS G12D	wt	*BRCA*wt/LOH-low	Sensitive	58.9	SD	86	SEROUS	HIGH GRADE	LGSC	Yes
14	KRAS G12V	wt	*BRCA*wt/LOH-low	Resistant	0.5	PD	55	SEROUS	HIGH GRADE	LGSC	Yes
15	KRAS G12V	LoF	*BRCA*wt/LOH-high	Sensitive	6.3	PD	75	SEROUS	HIGH GRADE	HGSC	No
16	KRAS G12D	LoF	*BRCA*wt/LOH-low	Sensitive	17.5	SD	74	SEROUS	HIGH GRADE	HGSC	No
17	NRAS Q61R	LoF	*BRCA*wt/LOH-high	Sensitive	6	SD	112	ENDOMETRIOID	HIGH GRADE	HGSC	No
18	KRAS G12V	LoF	*BRCA*mut	Sensitive	16.7	PR	515	SEROUS	HIGH GRADE	HGSC	No
19	KRAS Q61H	LoF	*BRCA*wt/LOH-low	Sensitive	10.9	PD	57	SEROUS	HIGH GRADE	HGSC	No
20	KRAS G12V	LoF	*BRCA*wt/LOH-low	Sensitive	49.7	PD	36	SEROUS	HIGH GRADE	HGSC	No
21	KRAS G12D	LoF	*BRCA*wt/LOH-low	Resistant	4.9	SD	55	SEROUS	HIGH GRADE	HGSC	No

Local pathology reports were used for determining patient eligibility for study enrollment.
[a]Mutation not detected in archival tissue.
BRCA, *BRCA1* or *BRCA2*; G1, 2, 3, grade 1, 2, 3; HGSC, high-grade serous carcinoma; LGSC, low-grade serous carcinoma; LoF, loss of function; LOH, loss of heterozygosity; mut, mutant; NA, not available; PFI, platinum-free interval; PFS, progression-free survival; PD, progressive disease; PR, partial response; SD, stable disease; wt, wild type.

Supplementary Table S13 | Primer Sequences for Methylation Analysis

Assay	Target	Sense	Antisense	Probe	Label
MSP	Methylated *BRCA1*	5'-TCGTGGTAACGGAAAAGCGC-3'	5'-AAATCTCAACGAACTCACGCCG-3'	NA	NA
MSP	Unmethylated *BRCA1*	5'-TTGGTTTTTGTGGTAATGGAAAAGTGT-3'	5'-CAAAAAATCTCAACAAACTCACACCA-3'	NA	NA
MSP	Methylated *RAD51C*	5’-TGTAAGGTTCGGAGTTTCGTGC-3’	5’-TCGCTAAAACGTACGACGTAACG-3’	NA	NA
MSP	Unmethylated *RAD51C*	5’-GTGTAAAGTTGTAAGGTTTGGAGTTTTGTGTG-3’	5’-CACACACCCTCACTAAAACATACAACATAACA-3’	NA	NA
MS-ddPCR	Methylated *BRCA1*	5'-GCGggaattaTagataaattaaaaTtg-3'	5’-tAtccccCGtccaAAaaAtctca-3’	5'-ActcacgccgcgcaA-3'	6-FAM
MS-ddPCR	Unmethylated *BRCA1*	5'-GCGggaattaTagataaattaaaaTtg-3'	5’-tAtccccCGtccaAAaaAtctca-3’	5'-ActcaCAcCACAcaAtc-3'	VIC
MS-ddPCR, methylation-sensitive digital droplet polymerase chain reaction; MS-PCR, methylation-specific polymerase chain reaction; NA, not applicable.					

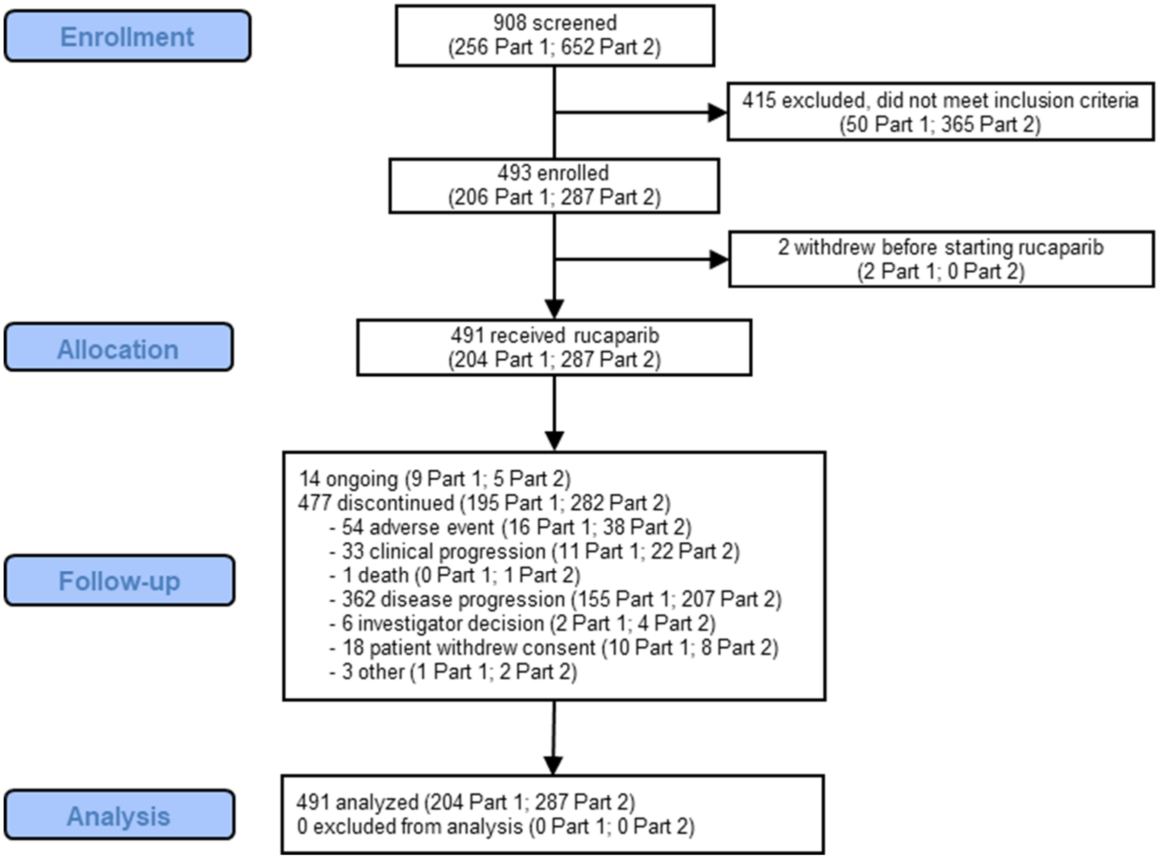

Supplementary Fig. S1 | CONSORT diagram of patient enrollment.

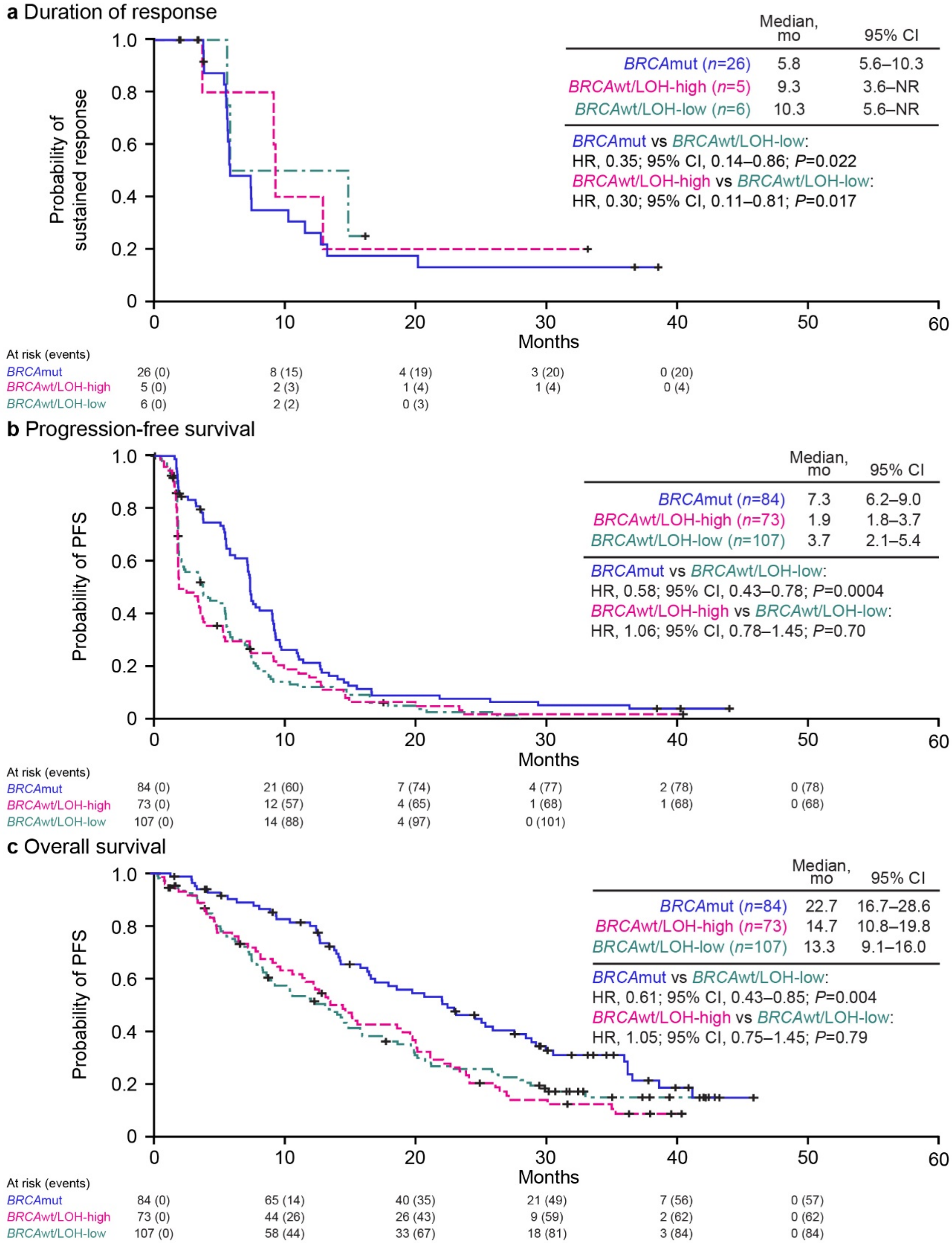

Supplementary Fig. S2 | (a) Duration of response, and (b, c) secondary endpoints by prespecified HRD subgroup in ARIEL2 Part 2. A prespecified cutoff of 18% was used for LOH; *P* values were computed using a Cox proportional hazard model. BRCA, *BRCA1* or *BRCA2*; CI,

confidence interval; HR, hazard ratio; HRD, homologous recombination deficiency; LOH, loss of heterozygosity; mut, mutated; wt, wild-type.

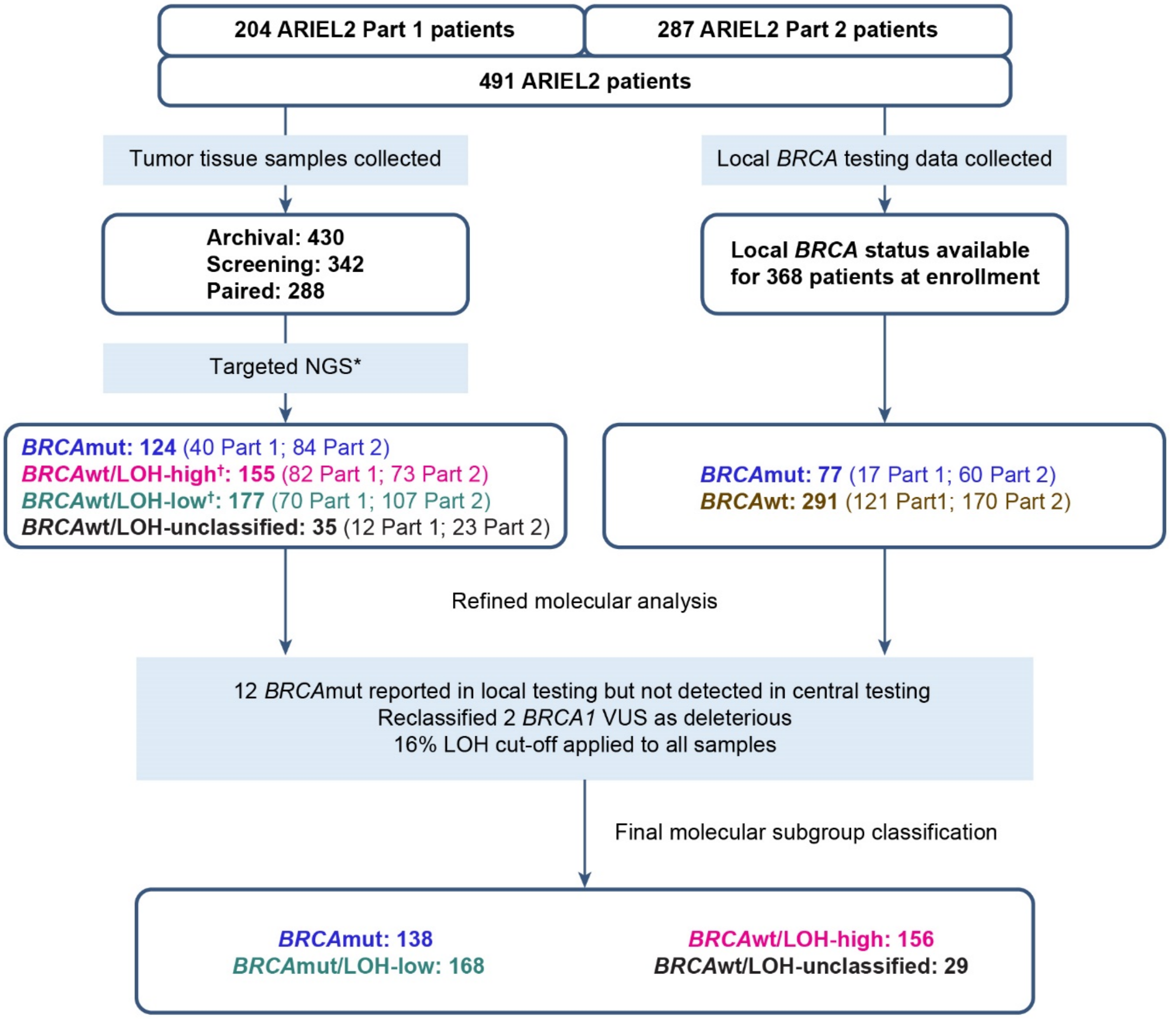

Supplementary Fig. S3 | Samples collection for molecular analyses from patients in ARIEL2 Parts 1 and 2. *Targeted NGS data only was used for all pre-specified analyses. †LOH was assigned based on screening sample when available; prespecified LOH cut-offs of 14% for ARIEL2 Part 1 and 18% for ARIEL2 Part 2 were used for all prespecified end-point analyses. BRCA, *BRCA1* or *BRCA2*; LOH, loss of heterozygosity; mut, mutated; NGS, next generation sequencing; VUS, variants of unknown significance; wt, wild-type.

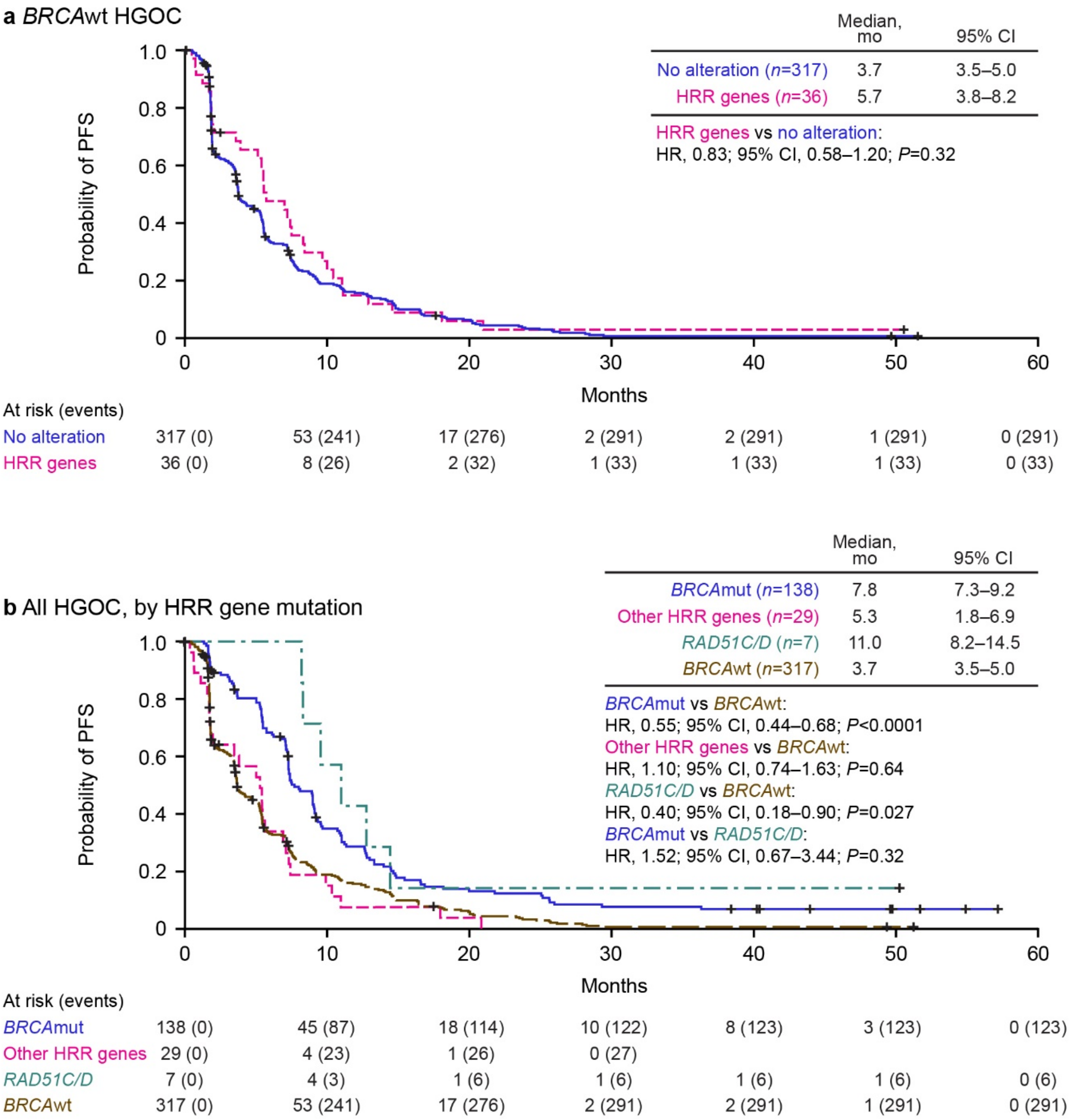

Supplementary Fig. S4 | PFS by HRR gene mutation. (a) PFS for patients with *BRCA*wt HGOC with (magenta) or without (blue) mutations in other HRR genes. **(b)** PFS in patients with HGOC with *BRCA* mutation (blue), *BRCA*wt HGOC with *RAD51C/D* mutation (green), *BRCA*wt HGOC with other HRR-gene mutation (magenta), and *BRCA*wt HGOC without any HRR-gene mutations (brown). *P* values were computed using a Cox proportional hazard model. BRCA, *BRCA1* or

BRCA2; CI, confidence interval; HGOC, high-grade ovarian carcinoma; HR, hazard ratio; HRR, homologous recombination repair; mut, mutated; PFS, progression-free survival; wt, wild-type.

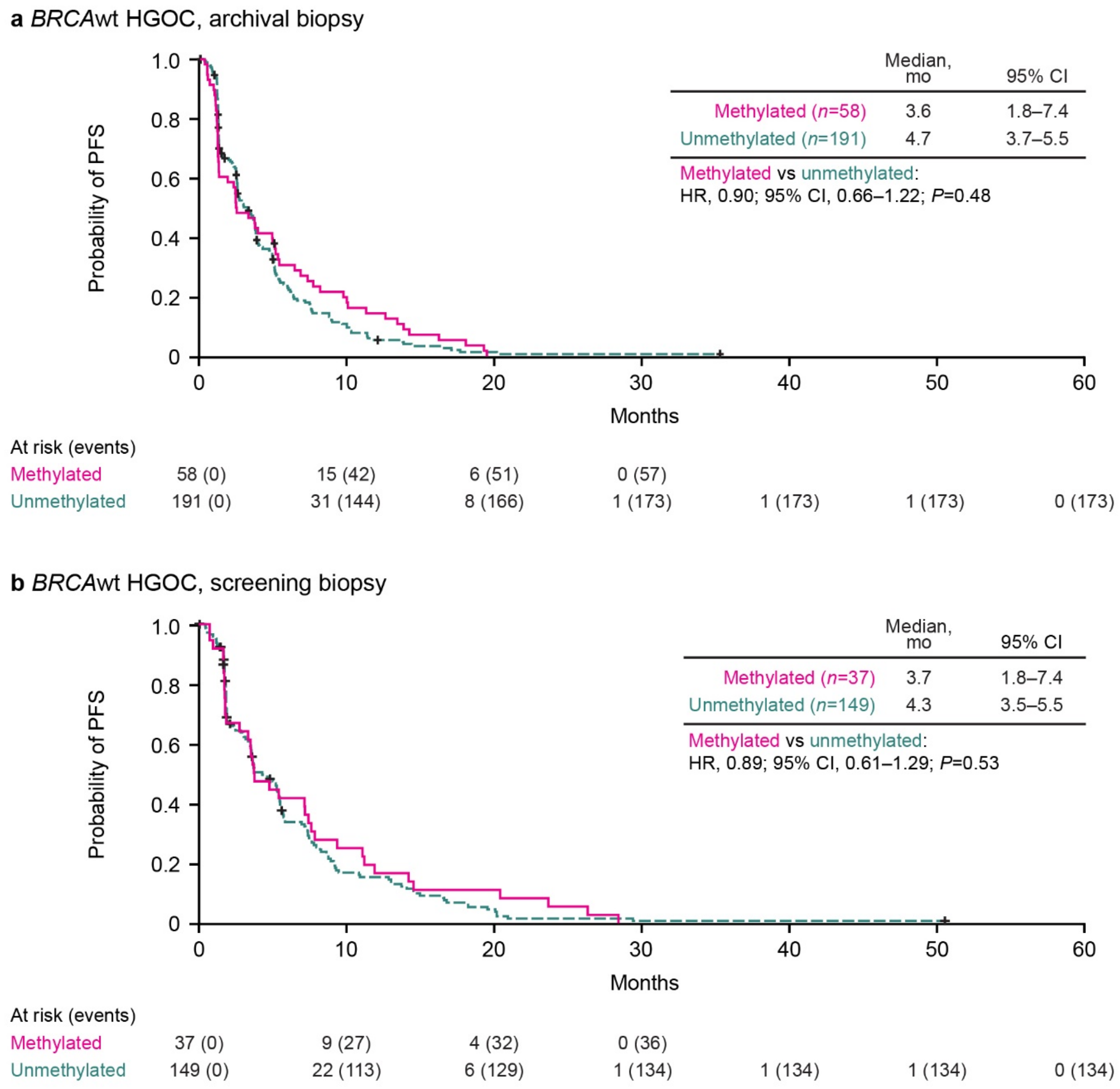

Supplementary Fig. S5 | PFS is similar in HGOC with *BRCA1/RAD51C* promoter methylation as determined by MSP analysis (without taking into account quantitative nature of methylation) and in those without detectable methylation. (a) PFS in patients with *BRCA*wt HGOC with (magenta) or without (teal) detectable *BRCA1* or *RAD51C* promoter methylation in archival pre-platinum tissue. **(b)** PFS in patients with *BRCA*wt HGOC with (magenta) or without (teal) detectable *BRCA1* or *RAD51C* promoter methylation in screening post-platinum biopsy. *P* values were computed using a Cox proportional hazard model. BRCA, *BRCA1* or *BRCA2*; CI, confidence

interval; HGOC, high-grade ovarian carcinoma; HR, hazard ratio; MSP, methylation-specific polymerase chain reaction; PFS, progression-free survival; wt, wild-type.

a HGOC with known *BRCA1* methylation status, archival biopsy

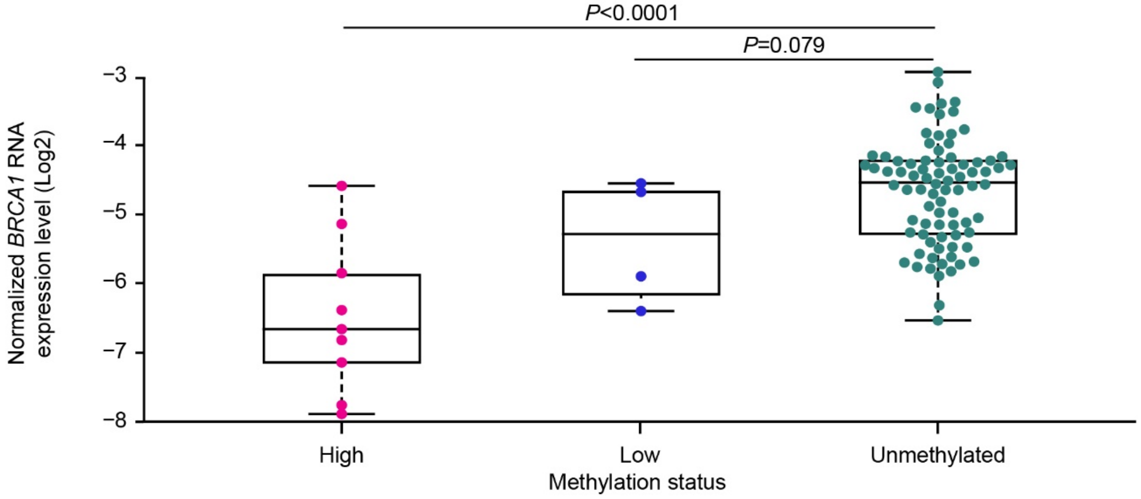

b HGOC by mutation or methylation status, screening biopsy

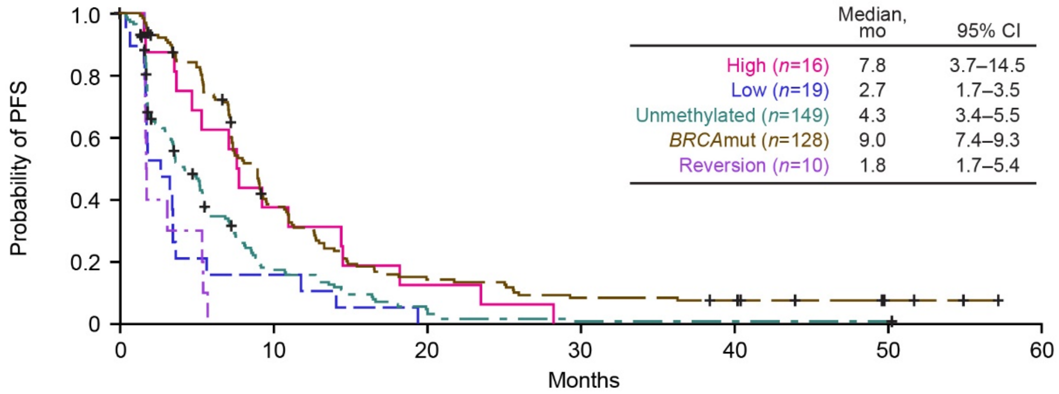

At risk (events)							
High	16 (0)	6 (10)	2 (14)	0 (16)			
Low	19 (0)	3 (16)	0 (19)				
Unmethylated	149 (0)	22 (112)	7 (127)	1 (133)	1 (133)	1 (133)	0 (133)
*BRCA*mut	128 (0)	45 (77)	18 (104)	10 (112)	8 (113)	3 (113)	0 (113)
Reversion	10 (0)	0 (10)					

Pairwise comparison	HR	95% CI	*P* value	Pairwise comparison	HR	95% CI	*P* value
High vs Low	0.40	0.19–0.80	0.01	*BRCA*mut vs Low	0.32	0.20–0.53	<0.0001
High vs Unmethylated	0.61	0.36–1.04	0.067	Low vs Reversion	0.73	0.33–1.62	0.44
*BRCA*mut vs High	0.84	0.50–1.42	0.52	*BRCA*mut vs Unmethylated	0.50	0.38–0.64	<0.0001
High vs Reversion	0.20	0.07–0.56	0.002	Reversion vs Unmethylated	0.43	0.23–0.84	0.013
Low vs Unmethylated	1.49	0.92–2.42	0.10	*BRCA*mut vs Reversion	0.11	0.05–0.23	<0.0001

Supplementary Fig. S6 | *BRCA1* RNA expression levels and association between methylation levels by quantitative MS-ddPCR and PFS in ARIEL2. (a) *BRCA1* RNA expression levels in HGOC with high, low, or no *BRCA1* methylation (unmethylated). Archival tumor specimens were

available for Nanostring assay to determine the *BRCA1* RNA expression level for 95 ARIEL2 patients with known archival *BRCA1* methylation status, determined by MS-ddPCR: 9 high methylation, 4 low methylation, and 82 unmethylated. Boxplots show median, quartiles, minimum, and maximum expression levels within each group. *P* values based on two-sided Wilcoxon rank sum tests. No multiple comparisons adjustment was performed. (b) Association between screening, post-platinum methylation levels determined by quantitative MS-ddPCR and PFS in ARIEL2. Kaplan-Meier plots showing PFS in ARIEL2 patients with HGOC and high (blue), low (red), or no methylation (unmethylated, green), or harboring a *BRCA* mutation (brown) or a *BRCA* reversion mutation (purple) in pretreatment biopsy. *P* values were computed using a Cox proportional hazard model. BRCA, *BRCA1* or *BRCA2*; CI, confidence interval; HGOC, high-grade ovarian carcinoma; HR, hazard ratio; MS-ddPCR, methylation-sensitive digital droplet polymerase chain reaction; mut, mutated; PFS, progression-free survival.

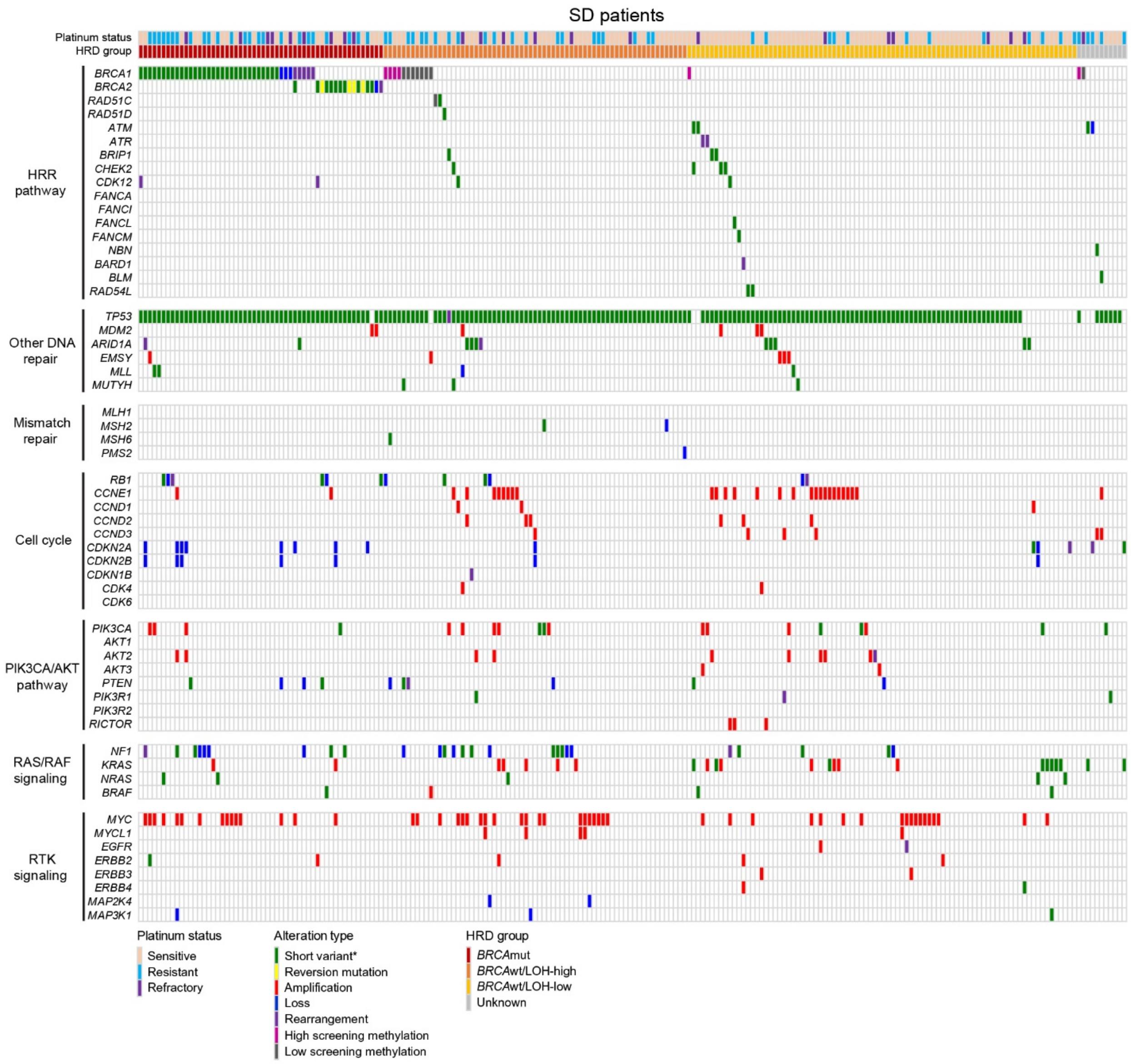

Supplementary Fig. S7 | Genetic and epigenetic alteration landscape of HGOC from ARIEL2 patients with confirmed best response of SD. Methylation levels shown are at screening. *Short variant include nonsense, missense, frameshift and splice site alterations. All reported alterations are deleterious or likely deleterious. HRD, homologous recombination deficiency; HRR, homologous recombination repair; LOH, loss of heterozygosity; mut, mutated; SD, stable disease; wt, wild-type.

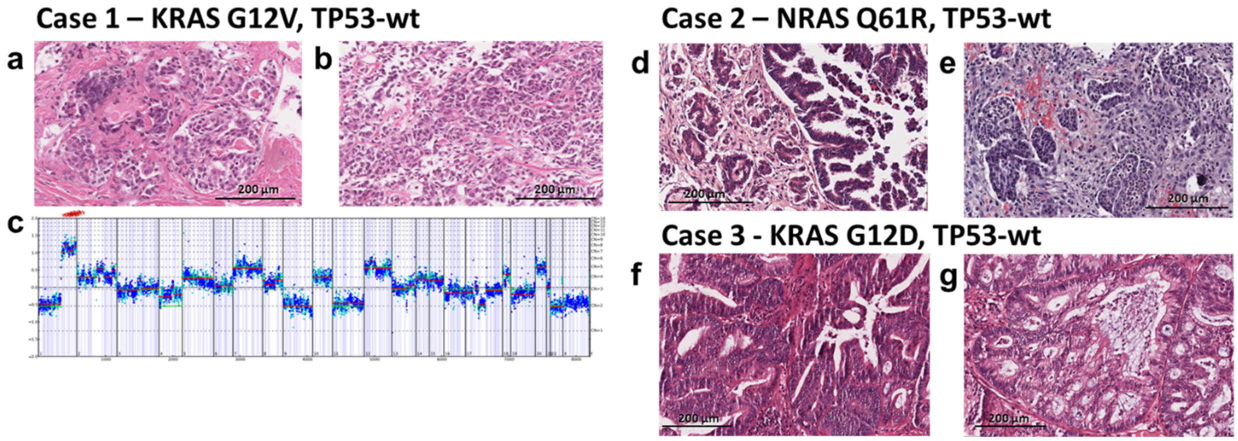

Supplementary Fig. S8 | Representative cases in which diagnosis was revised for ovarian carcinomas with *KRAS*/*NRAS*/*BRAF* activating mutation and no *TP53* alteration (*TP53*-wt).

Case 1 (Patient #1 in Table S12) was reclassified as mesonephric-like carcinoma based on morphological features characterized by **(a)** tubular and glandular architecture with prominent luminal secretions, **(b)** solid and nested morphology, combined and integrated with tumor genomics harboring *KRAS* G12V, and **(c)** gain of chromosome 1q, 10, and 12 and loss of 1p. Case 2 (Patient #4 in Table S12) was reclassified as low-grade serous carcinoma based on morphological features of both **(d)** archival and **(e)** screening samples demonstrating papillary architecture and invasive structures with retraction artifact, low-grade nuclear atypia, and low mitotic activity. Case 3 (Patient #10 in Table S12) was revised to low-grade, Grade 1 endometrioid adenocarcinoma based on morphological features, demonstrating cribriform architecture, low-grade nuclear atypia, no solid areas, **(f)** columnar cell morphology, and **(g)** mucinous differentiation. Both archival and screening tissue H&E slides were available for at least two patients each for mesonephric-like carcinomas, low-grade serous carcinomas, and endometrioid adenocarcinomas, and the reclassification was confirmed in both samples. H&E, hemotoxylin and eosin; wt, wild-type.

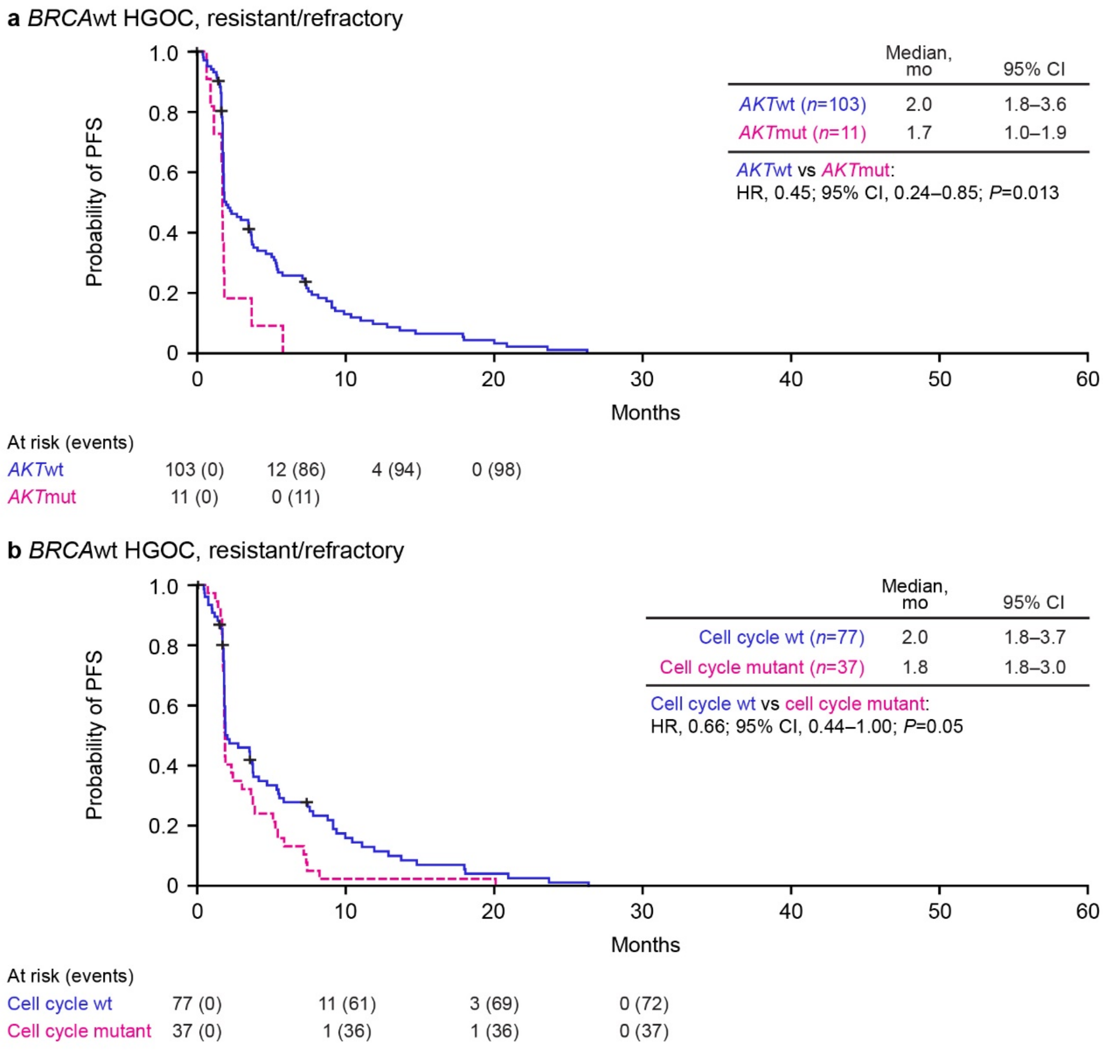

Supplementary Fig. S9 | Alterations in the *AKT* genes and the cell cycle pathway modulate rucaparib response in platinum-resistant or refractory patients. (a) Kaplan-Meier plot showing progression-free survival in patients with *BRCA*wt platinum-resistant/refractory HGOC with (magenta) or without (blue) alterations in the *AKT1/2/3* genes in pretreatment biopsies. **(b)** Kaplan-Meier plot showing progression-free survival in ARIEL2 patients with *BRCA*wt platinum-resistant/refractory HGOC (magenta) or without (blue) alterations in cell cycle genes (*CCND1*, *CCND2*, *CCND3*, *CCNE1*, *CDKN1B*, *CDKN2A*, *CDKN2B*, *CDK4*, *CDK6*) in pretreatment biopsies. *P* values were computed using a Cox proportional hazard model. CI, confidence interval; HGOC, high-grade ovarian carcinoma; HR, hazard ratio; mut, mutated; wt, wild-type.

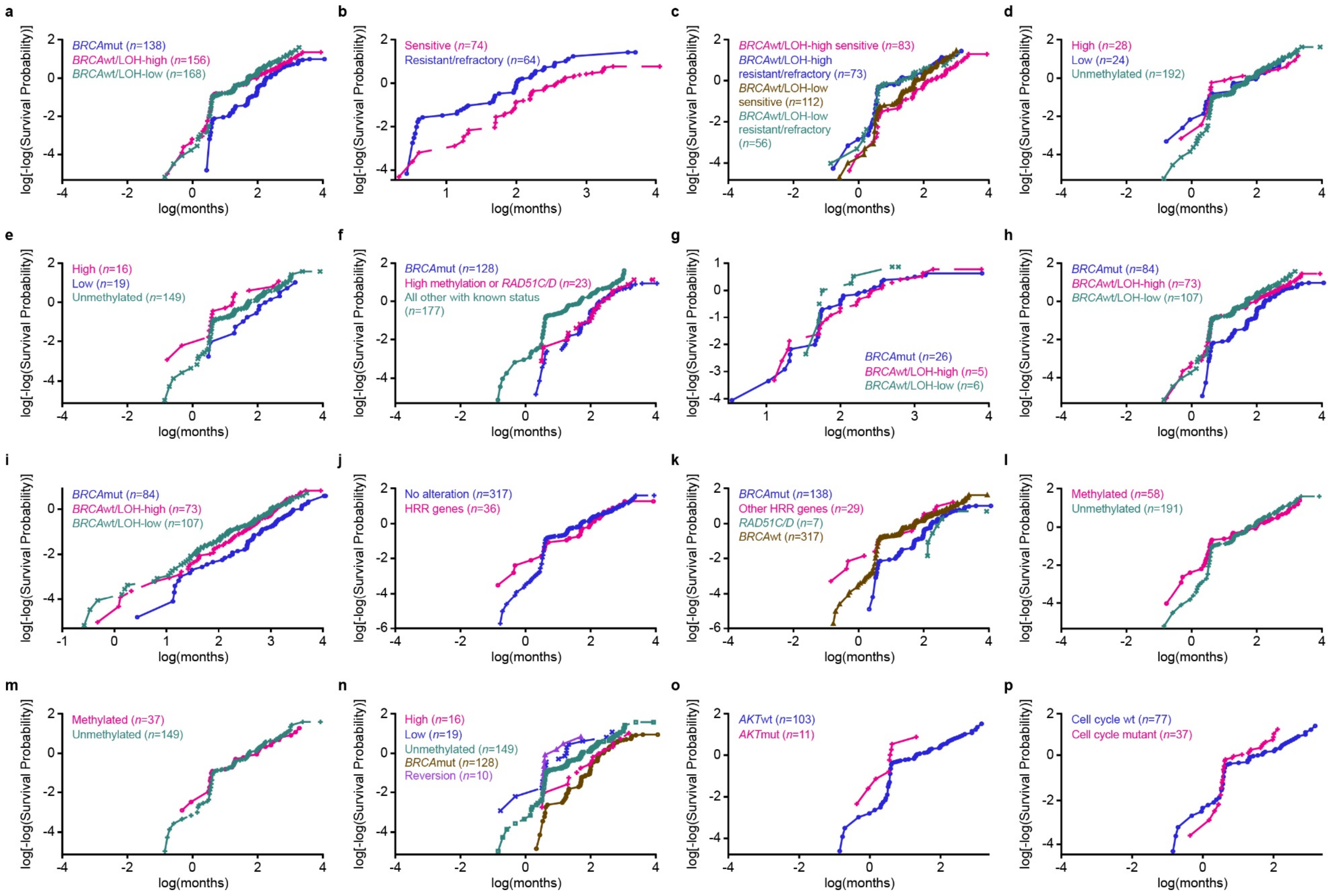

Supplementary Fig. S10 | Log-log plots used to visually assess the proportional hazards assumption for survival analyses presented in this manuscript. (a) Log-log plot related to Fig. 1a; **(b)** Log-log plot related for Fig. 1b; **(c)** Log-log plot related to Fig. 1c;

(d) Log-log plot related to Fig. 3a; **(e)** Log-log plot related to Fig. 3b; **(f)** Log-log plot related to Fig. 5; **(g)** Log-log plot related to Supplementary Fig. S2a; **(h)** Log-log plot related to Supplementary Fig. S2b; **(i)** Log-log plot related to Supplementary Fig. S2c; **(j)** Log-log plot related to Supplementary Fig. S4a; **(k)** Log-log plot related to Supplementary Fig. S4b; **(l)** Log-log plot related to Supplementary Fig. S5a; **(m)** Log-log plot related to Supplementary Fig. S5b; **(n)** Log-log plot related to Supplementary Fig. S6b; **(o)** Log-log plot related to Supplementary Fig. S9a; **(p)** Log-log plot related to Supplementary Fig. S9b. BRCA, *BRCA1* or *BRCA2*, HRR, homologous recombination repair; LOH, loss of heterozygosity; mut, mutated; wt, wild-type.
