# Supplementary material for: Molecular and clinical determinants of response and resistance to rucaparib for recurrent ovarian cancer treatment in ARIEL2 (Parts 1 and 2)
Source: Nat Commun. 2021 May 3;12:2487. doi: 10.1038/s41467-021-22582-6 (PMC8093258; doi:10.1038/s41467-021-22582-6)
Supplement: Supplementary file 2 — Reporting Summary [file 41467_2021_22582_MOESM2_ESM.pdf]

nature research

Corresponding author(s): Elizabeth M. Swisher

Last updated by author(s): Feb 24, 2021

Reporting Summary

Nature Research wishes to improve the reproducibility of the work that we publish. This form provides structure for consistency and transparency in reporting. For further information on Nature Research policies, see our Editorial Policies and the Editorial Policy Checklist.

Statistics

For all statistical analyses, confirm that the following items are present in the figure legend, table legend, main text, or Methods section.

n/a	Confirmed	
[ ]	[x]	The exact sample size (*n*) for each experimental group/condition, given as a discrete number and unit of measurement
[x]	[ ]	A statement on whether measurements were taken from distinct samples or whether the same sample was measured repeatedly
[ ]	[x]	The statistical test(s) used AND whether they are one- or two-sided<br>*Only common tests should be described solely by name; describe more complex techniques in the Methods section.*
[x]	[ ]	A description of all covariates tested
[ ]	[x]	A description of any assumptions or corrections, such as tests of normality and adjustment for multiple comparisons
[ ]	[x]	A full description of the statistical parameters including central tendency (e.g. means) or other basic estimates (e.g. regression coefficient) AND variation (e.g. standard deviation) or associated estimates of uncertainty (e.g. confidence intervals)
[ ]	[x]	For null hypothesis testing, the test statistic (e.g. *F*, *t*, *r*) with confidence intervals, effect sizes, degrees of freedom and *P* value noted<br>*Give P values as exact values whenever suitable.*
[x]	[ ]	For Bayesian analysis, information on the choice of priors and Markov chain Monte Carlo settings
[x]	[ ]	For hierarchical and complex designs, identification of the appropriate level for tests and full reporting of outcomes
[x]	[ ]	Estimates of effect sizes (e.g. Cohen's *d*, Pearson's *r*), indicating how they were calculated

Our web collection on statistics for biologists contains articles on many of the points above.

Software and code

Policy information about availability of computer code

Data collection	Data collection and formatting was performed using SAS v9.4 and Excel 2016.
Data analysis	Data analysis was performed using SAS v9.4 and Excel 2016.

For manuscripts utilizing custom algorithms or software that are central to the research but not yet described in published literature, software must be made available to editors and reviewers. We strongly encourage code deposition in a community repository (e.g. GitHub). See the Nature Research guidelines for submitting code & software for further information.

Data

Policy information about availability of data

All manuscripts must include a data availability statement. This statement should provide the following information, where applicable:

- Accession codes, unique identifiers, or web links for publicly available datasets
- A list of figures that have associated raw data
- A description of any restrictions on data availability

COPY PASTE FROM MANUSCRIPT

Requests for de-identified datasets for the results reported in this publication will be made available to qualified researchers following submission of a methodologically sound proposal to medinfo@clovisoncology.com. Data will be made available for such requests following online publication of this article and for 1 year thereafter in compliance with applicable privacy laws, data protection, and requirements for consent and anonymization. Data will be provided by Clovis Oncology. The redacted protocol for the ARIEL2 clinical study is available on thelancet.com: https://ars.els-cdn.com/content/image/1-s2.0-S1470204516305599-mmc1.pdf. Clovis Oncology does not share identified participant data or a data dictionary.

Field-specific reporting

Please select the one below that is the best fit for your research. If you are not sure, read the appropriate sections before making your selection.

- [x] Life sciences
- [ ] Behavioural & social sciences
- [ ] Ecological, evolutionary & environmental sciences

For a reference copy of the document with all sections, see nature.com/documents/nr-reporting-summary-flat.pdf

Life sciences study design

All studies must disclose on these points even when the disclosure is negative.

Sample size	Sample size calculations were previously reported in Swisher, et al. Lancet Oncol. 18, 75-87 (2017).
Data exclusions	No data exclusions.
Replication	The data in these analyses were derived from a clinical trial; therefore, experimental replication was not feasible.
Randomization	Allocation was not random. Patients were grouped based on molecular characteristics of their tumor and a Cox proportional hazard model was used to summarize endpoints and make comparisons between molecular subgroups.
Blinding	ARIEL2 was designed as a single-arm, open-label, nonrandomized, phase 2 study of rucaparib; therefore blinding was not performed.

Reporting for specific materials, systems and methods

We require information from authors about some types of materials, experimental systems and methods used in many studies. Here, indicate whether each material, system or method listed is relevant to your study. If you are not sure if a list item applies to your research, read the appropriate section before selecting a response.

Materials & experimental systems

n/a	Involved in the study	
x		Antibodies
x		Eukaryotic cell lines
x		Palaeontology and archaeology
x		Animals and other organisms
	x	Human research participants
	x	Clinical data
x		Dual use research of concern

Methods

n/a	Involved in the study	
x		ChIP-seq
x		Flow cytometry
x		MRI-based neuroimaging

Human research participants

Policy information about studies involving human research participants

Population characteristics	In ARIEL2, eligible patients were aged 18 years or older with histologically confirmed, relapsed, high-grade serous or Grade 2 or Grade 3 endometroid epithelial ovarian, fallopian tube, or primary peritoneal cancer. Part 1 enrolled patients with relapsed HGOC who had received at least one prior platinum-based regimen and had platinum-sensitive disease (disease progression ≥6 months after last platinum). Part 2 enrolled patients with relapsed HGOC who had received three to four prior chemotherapies and had a treatment-free interval of more than 6 months following first-line chemotherapy. Patients in Part 2 could be platinum sensitive, platinum resistant (disease progression <6 months after last platinum, with best response other than PD), or platinum refractory (best response of PD on last platinum with progression-free interval <2 months).
Recruitment	Patients were screened and enrolled at local study sites and institutions across 64 sites in 6 countries (Australia, Canada, France, Spain, the United Kingdom, and the United States) to ensure enrollment was competitive and representative. The full list of study sites has been previously published (Swisher, et al. Lancet Oncol. 18, 75-87 [2017] - page 3 of Supplementary Info).<br><br>Patients were screened by trained personnel, and principal investigators at each site were responsible for evaluating and confirming eligibility. Any patient who met the inclusion/exclusion criteria as detailed in the study protocol was considered for enrollment, therefore, to the best of our knowledge, there was no selection bias during recruitment.
Ethics oversight	The study was approved by national or local institutional review boards, as appropriate at each site:<br><br>Alberta Cancer Research Ethics Committee (ACREC), BC Cancer Agency - Vancouver Cancer Centre, CEIm Hospital Clínico Universitario de Valencia, Comité d'Éthique de la recherche du CHUM, Dana-Faber Cancer Institute Office For Human Research Studies, Fox Chase Cancer Center IRB, France CPP No., Fred Hutchinson Cancer Research Center Institutional Review Office, Health Research Ethics Board of Alberta Cancer Committee, Jewish General Hospital Research Ethics committee, Johns Hopkins Institutional Review Board, Massachusetts General Hospital Partners Human Research Committee, Mayo Clinic IRB, Melbourne Health HREC, Melbourne Health Office For Research, Memorial Sloan Kettering Cancer Center IRB/Privacy Board, Mercy Health HREC, Mission Health System IRB, Northern Sydney Local Health District Research Governance Office, NYU School of Medicine Institutional Review Board, Ontario Cancer Research Ethics Board, Orlando Regional Medical Center IRB, Quorum Review IRB (Now Advarra), Royal Brisbane and Women's Hospital Service District, Human Research Ethics Committee, Sir Charles Gairdner and Osborne Park Health Care Group, Human Research Ethics Committee, South Eastern Sydney Local Health District, Southern Adelaide Clinical Human Research Ethics Committee, Stanford University Institutional Review Board, The University of Texas MD Anderson Cancer Center IRB, Texas Oncology Austin Brain Tumor Center, UBC BC Cancer Agency Research Ethics Board, UCLA Office of Human Research Protection Program, UC San Diego Human Research Protections Program, University of California San Francisco Human Research Protection Program, University of Miami Human Subject Research Office, University of Oklahoma Health Sciences Center IRB, University of Pennsylvania IRB, Washington University in St. Louis Human Research Protection Office, West of Scotland Research Ethics Service, Western Institutional Review Boards, Western Sydney Local Health District Research Governance Office

Note that full information on the approval of the study protocol must also be provided in the manuscript.

Clinical data

Policy information about clinical studies

All manuscripts should comply with the ICMJEguidelines for publication of clinical research and a completedCONSORT checklist must be included with all submissions.

Clinical trial registration	NCT01891344
Study protocol	The redacted protocol for the ARIEL2 clinical study is available on thelancet.com: https://ars.els-cdn.com/content/image/1-s2.0-S1470204516305599-mmc1.pdf.
Data collection	Between October 2013 and August 2016, 491 patients were enrolled in ARIEL2 (Part 1, n=204; Part 2, n=287) at hospitals and clinics (64 sites in 6 countries [Australia, Canada, France, Spain, the United Kingdom, and the United States]). Data are presented through a visit cutoff of 01 Feb 2019.
Outcomes	The primary endpoint in Part 1 was PFS by predefined HRD subgroups. PFS was defined as the number of days from the first dose of study drug to disease progression by RECIST, as determined by the investigator, or death due to any cause, whichever occurred first.<br><br>In Part 2, the primary endpoint was ORR, defined as the proportion of patients achieving a best response of complete or partial response according to RECIST as assessed by the investigator by predefined HRD subgroups.<br><br>Secondary endpoints included the proportion of patients achieving an objective response (according to RECIST and GCIG CA-125 criteria), duration of response (according to RECIST), and overall survival.<br><br>The response (partial or complete response) by RECIST needed to be confirmed by a second assessment after at least 4 weeks. Duration of confirmed response (complete or partial response) was calculated from the initial date a response was detected to the first date of PD. Patients without a documented event of progression were censored on the date of their last adequate cancer assessment (ie, radiologic assessment) or date of response if no cancer assessments were performed. Overall survival was defined as the number of days from the date of first dose of study drug to the date of death (due to any cause). Patients without a known date of death were censored on the date the patient was last known to be alive.
